# Top 100 most-cited articles on apoptosis of non-small cell lung cancer over the past two decades: a bibliometrics analysis

**DOI:** 10.3389/fimmu.2024.1512349

**Published:** 2025-01-13

**Authors:** Leshi Ma, Jing Zhang, Zi Dai, Pei Liao, Jieshan Guan, Zhijie Luo

**Affiliations:** ^1^ First Clinical Medical College, Guangzhou University of Chinese Medicine, Guangzhou, China; ^2^ Department of Oncology, Chongqing Hospital, The First Affiliated Hospital of Guangzhou University of Chinese Medicine, Chongqing, China; ^3^ Department of Oncology, The First Affiliated Hospital of Guangzhou University of Chinese Medicine, Guangzhou, China; ^4^ Guangdong Clinical Research Academy of Chinese Medicine, Guangzhou, China; ^5^ Lingnan Medical Research Center, Guangzhou University of Chinese Medicine, Guangzhou, China; ^6^ Department of Oncology, Shenshan Hospital, The First Affiliated Hospital of Guangzhou University of Chinese Medicine, Shanwei, China

**Keywords:** non-small cell lung cancer, apoptosis, cited articles, bibliometrics, visual analysis

## Abstract

**Background:**

Recently there has been an increasing number of studies have explored apoptosis mechanisms in lung cancer (LC). However, no researchers have conducted a bibliometric analysis of the most cited articles in this field.

**Objective:**

To examine the top 100 most influential and cited publications on apoptosis in non-small cell lung cancer (NSCLC) from 2004 to 2023, summarizing research trends and key focus areas.

**Methods:**

This study utilized the Web of Science Core Database (WOSCC) to research NSCLC apoptosis from 2004 to 2023, using keyword selection and manual screening for article searches. Bibliometrix package of R software 4.3.1 was used to generate distribution statistics for the top ten institutions, journals and authors. Citespace6.2. R6 was used to create the visualization maps for keyword co-occurrence and clustering. VOSviewer1.6.19 was used to conduct cluster analysis of publishing countries (regions), with data exported to SCImago Graphica for geographic visualization and cooperation analysis. VOSviewer1.6.19 was used to produced co-citation maps of institutions, journals, authors, and references.

**Results:**

From 2004 to 2023, 13316 articles were retrieved, and the top 100 most cited were chosen. These were authored by 934 individuals from 269 institutions across 18 countries and appeared in 45 journals. Citations ranged from 150 to 1,389, with a median of 209.5. The most influential articles appeared in 2005 and 2007 (n=13). The leading countries (regions), institutions, journals and authors were identified as the United States (n=60), Harvard University (n=64), *CANCER RESEARCH* (n=15), SUN M and YANG JS (n=6). The top five keywords were “expression”, “activation”, “apoptosis”, “pathway” and “gefitinib”. This study indicates that enhancing apoptosis through circular RNA regulation and targeting the Nrf2 signaling pathway could become a key research focus in recent years.

**Conclusion:**

Apoptosis has been the subject of extensive research over many years, particularly in relation to its role in the pathogenesis, diagnosis, and treatment of NSCLC. This study aims to identify highly influential articles and forecast emerging research trends, thereby offering insights into novel therapeutic targets and strategies to overcome drug resistance. The findings are intended to serve as a valuable reference for scholars engaged in this field of study.

## 1 Introduction

Lung cancer (LC) is one of the most common cancers in the world and is the foremost cause of cancer-related mortality, characterized by high incidence and mortality rates (1). Non-small cell lung cancer (NSCLC) represents the predominant subtype of LC, comprising 80%-85% of cases (2). The occurrence of apoptosis plays an important role in the development of cancer, so it is particularly important to study the mechanism of apoptosis in NSCLC for therapeutic advancements. The mechanisms of apoptosis encompass endogenous pathways, which are activated by factors such as DNA damage, oxidative stress and a variety of other stress conditions, as well as extrinsic pathways, which are initiated by cell membrane proteins associated with death receptors (3, 4). Alterations in upstream regulatory factors within the two pathways may lead to the imbalance between cell proliferation and apoptosis (5).

Bibliometrics uses mathematical and statistical techniques to quantitatively and qualitatively assess publications within a given research domain, thereby facilitating the monitoring of research trends and serving as a diagnostic tool for the field (6, 7). Bibliometrics serves as a tool for assessing the current state of research, identifying research hotspots, and predicting future research trajectories (8). Citespace, a scientific visualization software package developed by Dr. Chaomei Chen from Drexel University, USA. It operates within a Java runtime environment and offers direct visual effects (9). VOSviewer is a software application designed for the construction and visualization of bibliometric maps. It also provides a viewer, which provides a detailed check on the scientific nature of cartography (10). The software is equipped with robust capabilities for conducting co-occurrence and co-citation analyses (11). Additionally, its data can be exported to SCImago Graphica for enhanced geographic visualization and collaboration analysis, thereby improving the aesthetic quality of the visual representations. Bibliometrix package of R software 4.3.1 serves as a comprehensive language and environment for performing statistical analyses and generating graphical representations of data (12). As a versatile analytical tool developed in R, Bibliometrix is particularly well-suited for the scientific mapping of bibliometric data (13).

The number of citations of an article serves as a significant metric for assessing its scholarly impact, with a high number of citations often indicating its recognition and validation within the academic community. Despite this, an analysis of the most influential articles in the domain of NSCLC apoptosis research has not yet been conducted. In this study, we selected the top 100 most frequently cited articles and analyzed their quality and characteristics in order to provide references for researchers.

## 2 Data and methods

### 2.1 Data source

**Figure 1:**
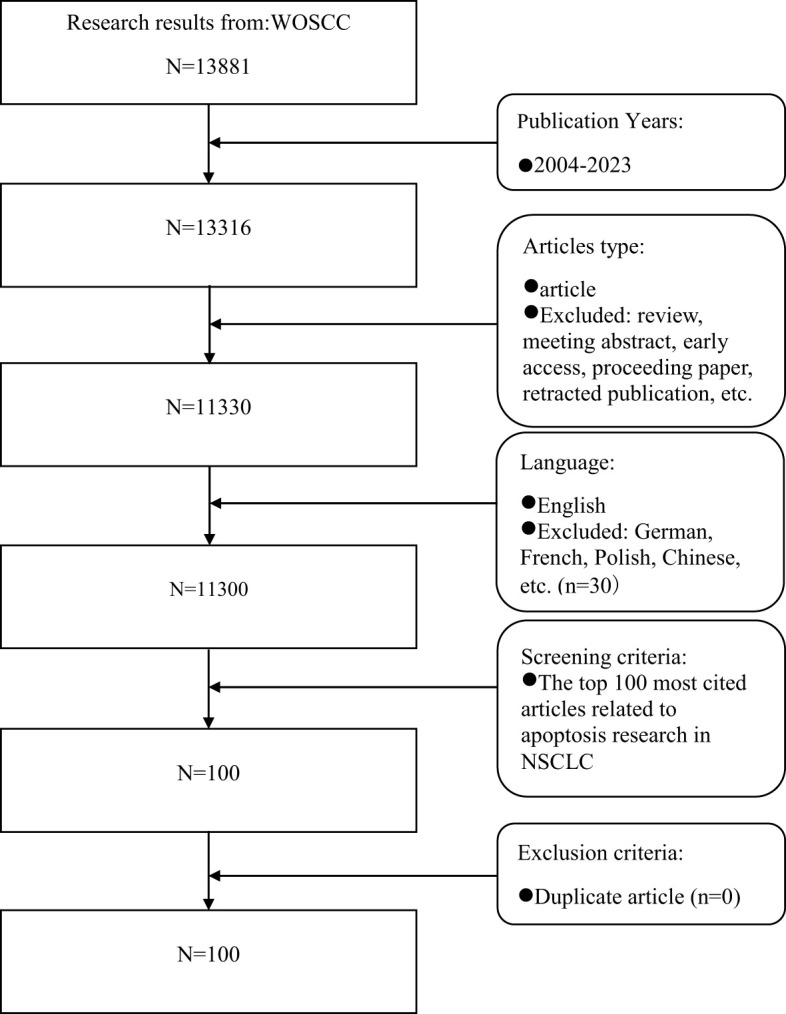
Article screening flow chart.

Related publications on NSCLC apoptosis research were retrieved from the Web of Science Core Database (WOSCC) on January 1, 2024, with a search formula of TS=(Carcinoma, Non-Small-Cell Lung OR Carcinoma, Non Small Cell Lung OR Carcinomas, Non-Small-Cell Lung OR Lung Carcinoma, Non-Small-Cell OR Lung Carcinomas, Non-Small-Cell OR Non-Small-Cell Lung Carcinomas OR Non-Small-Cell Lung Carcinoma OR Non Small Cell Lung Carcinoma OR Carcinoma, Non-Small Cell Lung OR Non-Small Cell Lung Carcinoma OR Non-Small Cell Lung Cancer OR Non small Cell Lung Cancer OR NSCLC) AND TS=(Apoptosis OR Classic Apoptosis OR Apoptosis, Classic OR Classic Apoptoses OR Programmed Cell Death, Type I OR Classical Apoptosis OR Apoptosis, Classical OR Apoptosis, Intrinsic Pathway OR Apoptoses, Intrinsic Pathway OR Intrinsic Pathway Apoptoses OR Intrinsic Pathway Apoptosis OR Apoptosis, Extrinsic Pathway OR Apoptoses, Extrinsic Pathway OR Extrinsic Pathway Apoptoses OR Extrinsic Pathway Apoptosis OR Caspase-Dependent Apoptosis OR Apoptosis, Caspase-Dependent OR Caspase Dependent Apoptosis OR Programmed Cell Death OR Cell Death, Programmed OR cell apoptosis OR cells apoptosis). The time span is 2004-2023, the article type is “article”, and the language of the article is “English”. The article was excluded if its title and abstract indicated inconsistency with the study’s topic. Subsequently, the 100 most-cited articles were selected for inclusion (see **Figure 1**). Complete records and references were exported and saved in plain text format for analysis using bibliometrics tools.

### 2.2 Visual analysis methods

The bibliometric visualization analysis was conducted using the Bibliometrix package of R software 4.3.1, Citespace6.2.R6, VOSviewer1.6.19 and SCImago Graphica 1.0.46. Citespace6.2.R6 was used to de-duplicate the exported plain text format, after which there were still 100 articles. The free statistics website (https://bibliometric.com/) was utilized to analyze publication time. Bibliometrix package of R software 4.3.1 was used to generate distribution statistics for the top ten institutions, journals, and authors. Additionally, Citespace version 6.2.R6 was used to create a visualization map for keyword co-occurrence and clustering, which served to elucidate the temporal distribution of research hotspots and to forecast future research trends. VOSviewer1.6.19 was used to conduct a cluster analysis of the publishing country (region). The resulting data were subsequently exported to SCImago Graphica for geographic visualization and collaboration analysis. Additionally, VOSviewer was used to construct co-citation maps of institutions, journals, authors, and references. These visualizations effectively highlight the articles that have significantly contributed to the advancement and development of research within this discipline.

## 3 Results

### 3.1 General data analysis

On January 1, 2024, a total of 13,881 articles were retrieved from WOSCC using the specified search formula. Following the screening process outlined in **Figure 1**, we identified the top 100 most frequently cited publications (14–113). Subsequently, we extracted detailed information on these publications, including their titles, journals, first authors, publication years, total citations and average annual citations (refer to the **Supplementary Materials** for further details). Among these selected articles, the number of citations ranged from 150 to 1,389, with a median of 209.5 and an average of 259.5 citations per year. In a seminal 2004 publication in the journal *SCIENCE*, SORDELLA R. et al. (2004) (14) published an article that has garnered the highest number of citations (1,389 times). Their research elucidates the selective activation of Akt and the signal transducer and activator of transcription (STAT) signaling pathways by mutated epidermal growth factor receptors (EGFRs). Furthermore, the study demonstrates that gefitinib effectively inhibits these pathways, thereby inducing apoptosis.

### 3.2 Analysis of publication time

**Figure 2:**
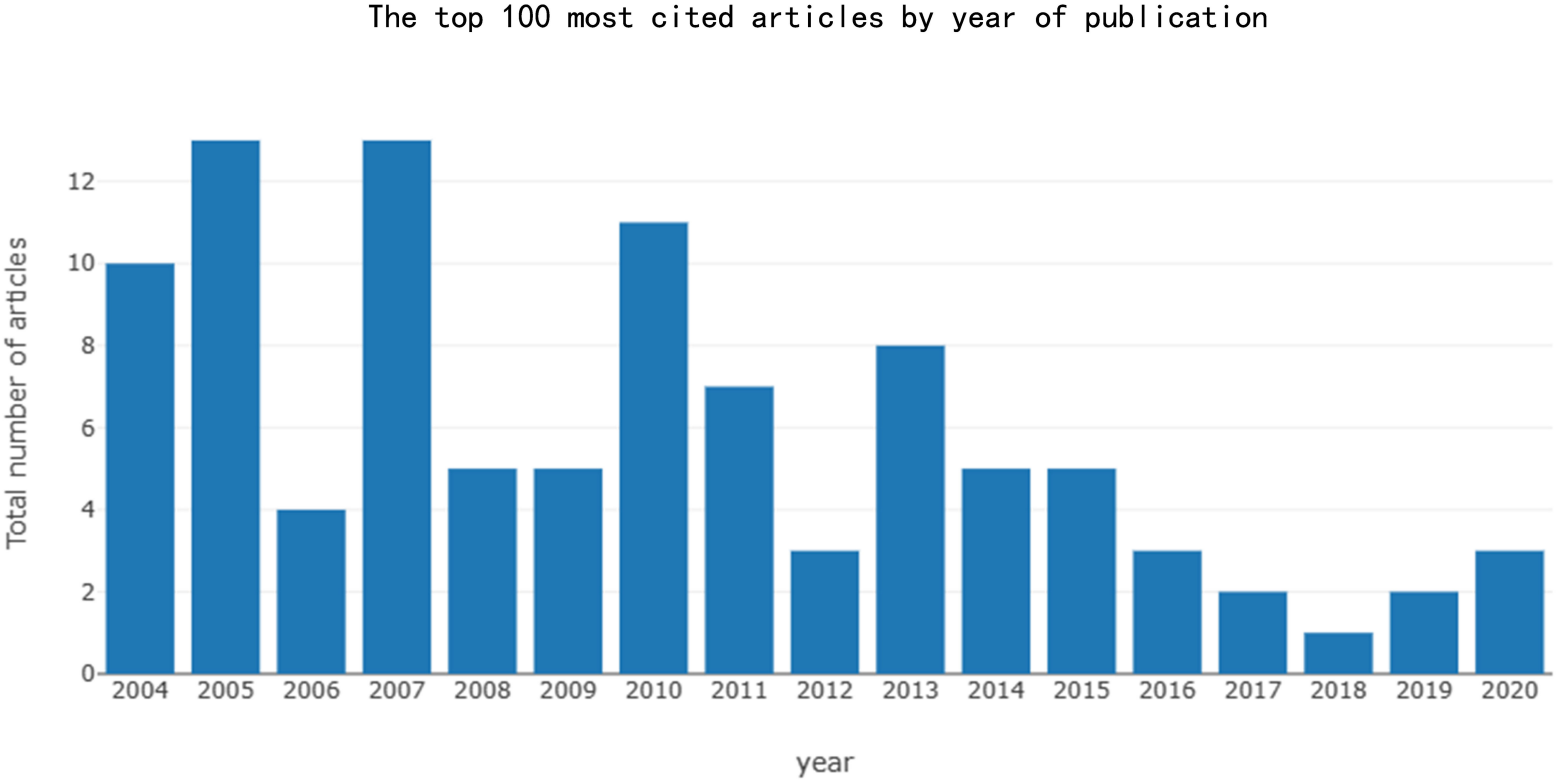
The top 100 most cited articles by year of publication.

Among the top 100 most-cited articles, the publication period spans from 2004 to 2020. There is a overall trend of decreasing article quantity over time, with an average annual growth rate in the number of publications of -7.25%. The most influential articles were produced in 2005 and 2007 (n=13) (see **Figure 2**). The earliest article was published by BRÖER LE. et al. (2004) (83) in *CANCER RESEARCH* journal in 2004, elucidated the pivotal role of the lysosomal protease cathepsin B in facilitating cell death. This study provided compelling evidence for a novel cathepsin B-dependent cell death pathway, which is induced by microtubule stabilizers in NSCLC cells.

### 3.3 Analysis of countries (regions)

**Figure 3:**
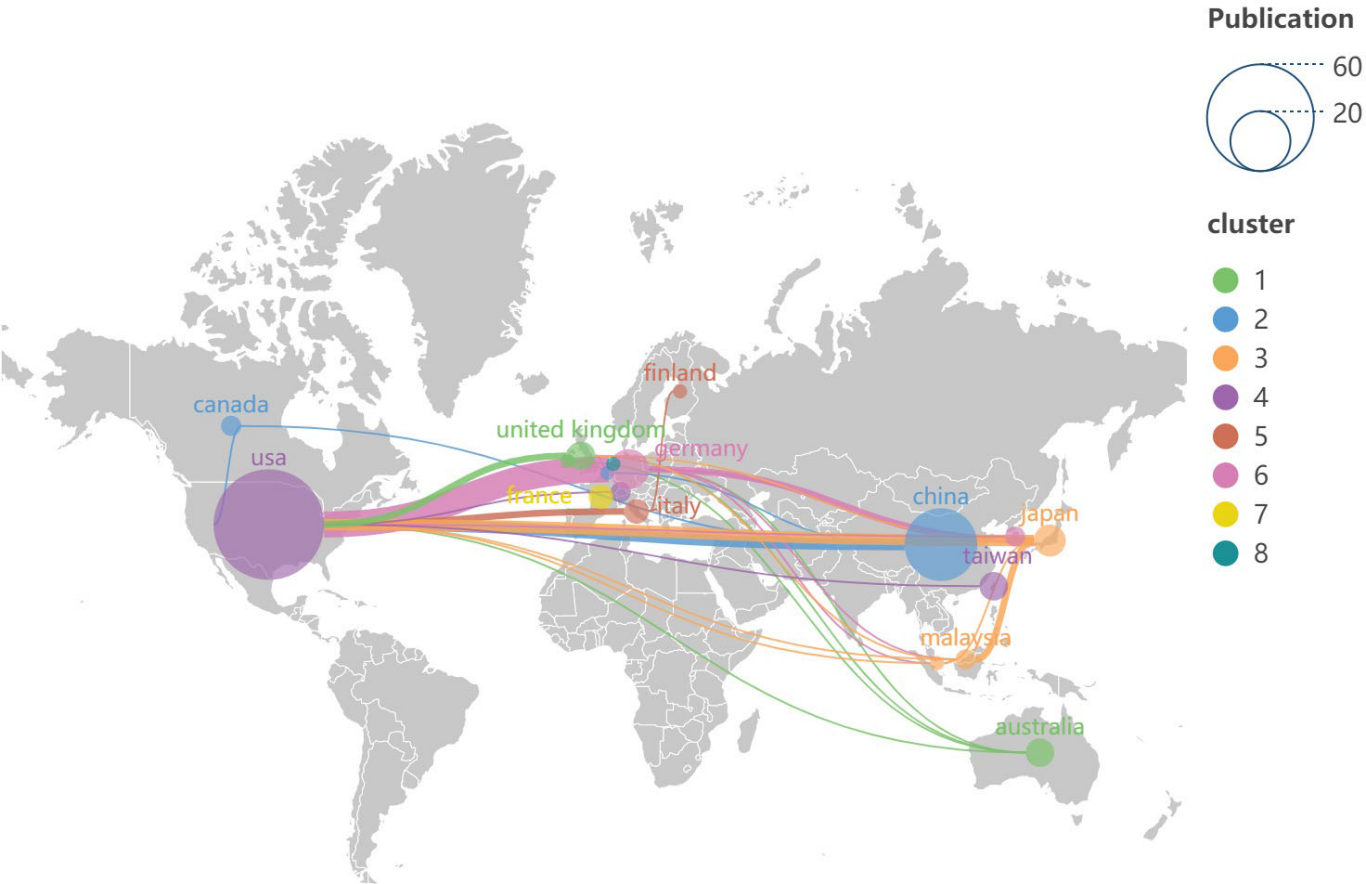
Distribution of countries and regions published on the world map. Each node symbolizes a country (region), with the size of the node indicating the volume of published articles originating from that country (region). The lines connecting nodes illustrate the degree of collaborative activity between the countries (regions). The color of each node corresponds to the cluster classification shown on the right side of the figure.

**Figure 4:**
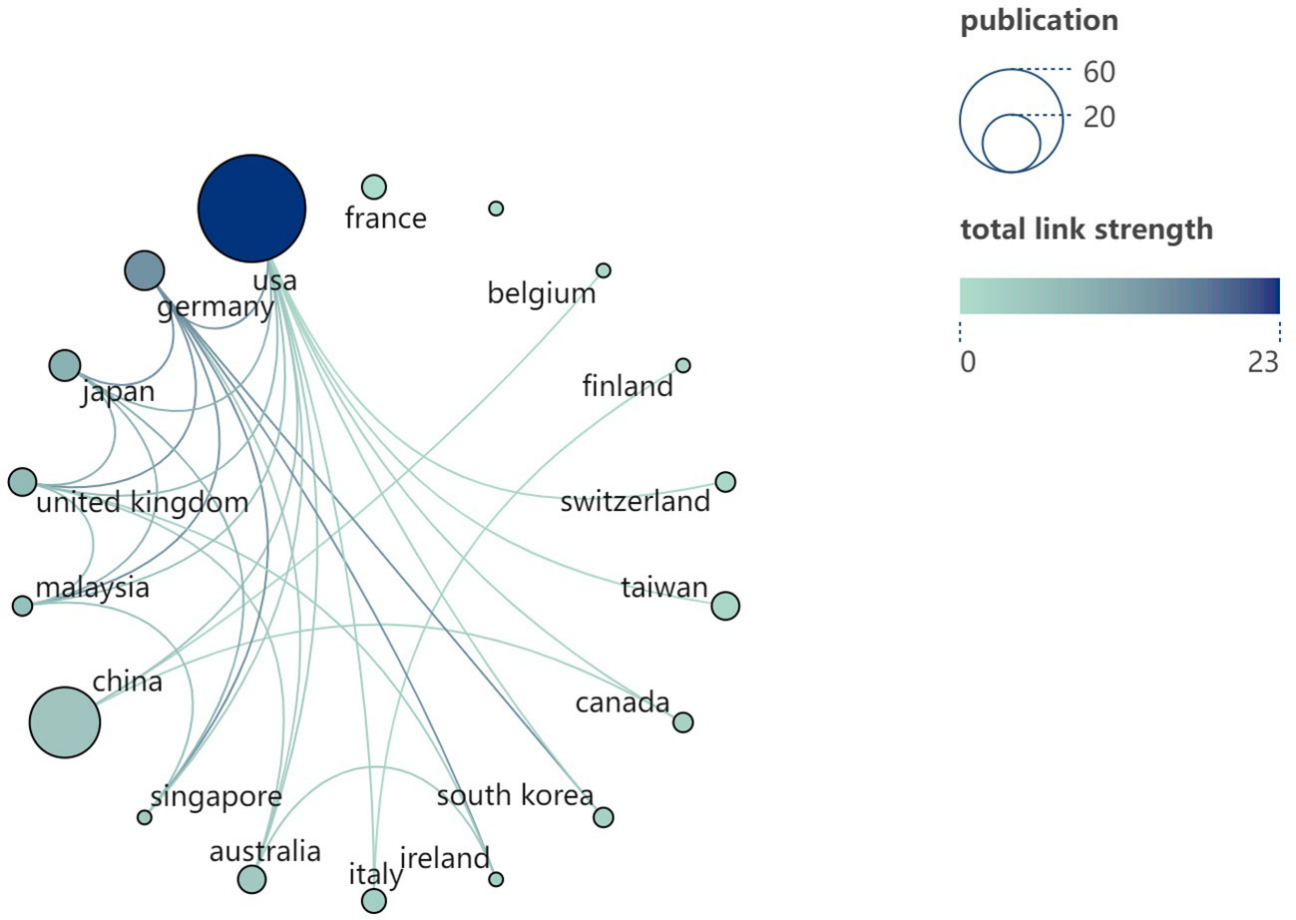
Cooperation relationship diagram between countries (regions). Each node symbolizes a country (region). The size of each node is indicative of the number of articles published within that country (region). The color of the node, as well as the connecting lines, signifies the aggregate number of interactions between the node in question and other nodes. Additionally, the color of the node correlates with the total link strength, as depicted on the right side of the visual representation.

Based on the data presented in **Table 1**, the top 100 most-cited articles originate from 18 countries (regions). The United States leads with the highest number of publications is from the United States (n=60), followed by China (n=26) and Germany (n=8). Notably, only the United States and China have published more than 20 articles among all the countries (region). The world map (**Figure 3**) shows the distribution of published countries (regions) is mainly in North America, East Asia and Western Europe. In contrast, Africa and South America have no top 100 most-cited articles in this field. This suggests a significant imbalance in the geographical distribution of influential research articles. Enhancing international collaboration is essential to advancing the development of this research domain. The United States not only leads in the volume of academic publications but also demonstrates the highest level of international collaboration. Furthermore, Germany, China and Italy also exhibit significant cooperation with other countries (regions) (see **Figure 4**).

**Table 1:** The top 10 countries (regions)/authors ranked by the number of articles.

Item	Rank	Name	Number of articles	Total citations	Citations/Year
Countries/regions	1	United States of America	60	15,665	301.20
	2	China	26	5,942	212.20
	3	Germany	8	735	245.30
	4	Japan	5	709	177.20
	5	Australia	4	447	447.00
	6	Taiwan	4	377	188.50
	7	England	4	318	318.00
	8	Italy	3	315	157.50
	9	France	3	297	279.00
	10	Canada	2	224	224.00
Authors	1	SUN M	6	1,531	145.98
	2	YANG JS	6	1,388	113.65
	3	ENGELMAN JA	6	1,519	86.02
	4	JÄNNE PA	4	1,562	115.01
	5	KONG R	4	833	82.15
	6	LIU XH	4	1,079	98.47
	7	MEYERSON M	4	1,519	82.63
	8	WANG ZX	4	857	76.89
	9	WONG KK	4	902	81.71
	10	XU TP	4	929	91.75

### 3.4 Analysis of institutions

**Figure 5:**
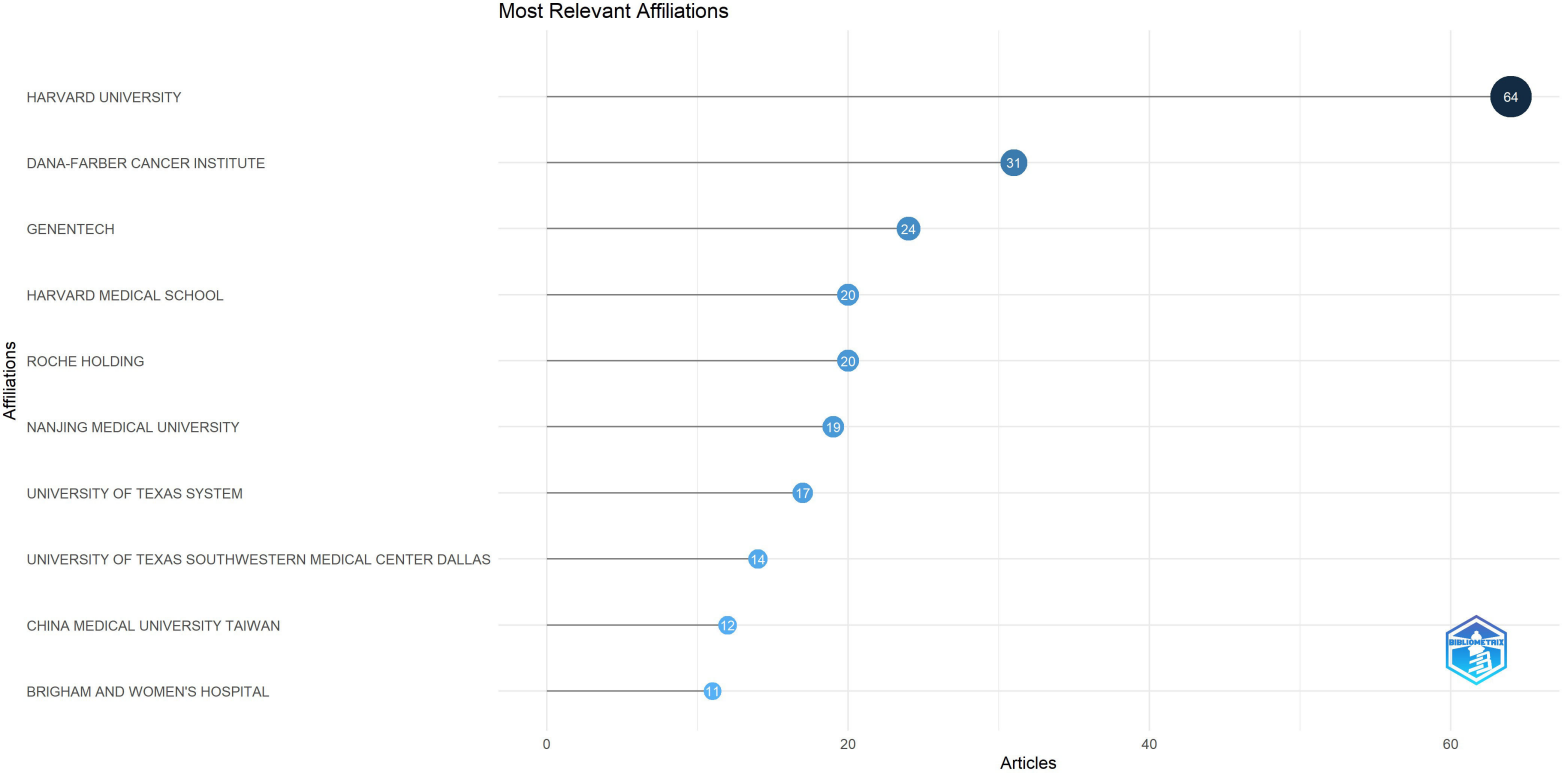
Top 10 institutions by the number of articles.

**Figure 6:**
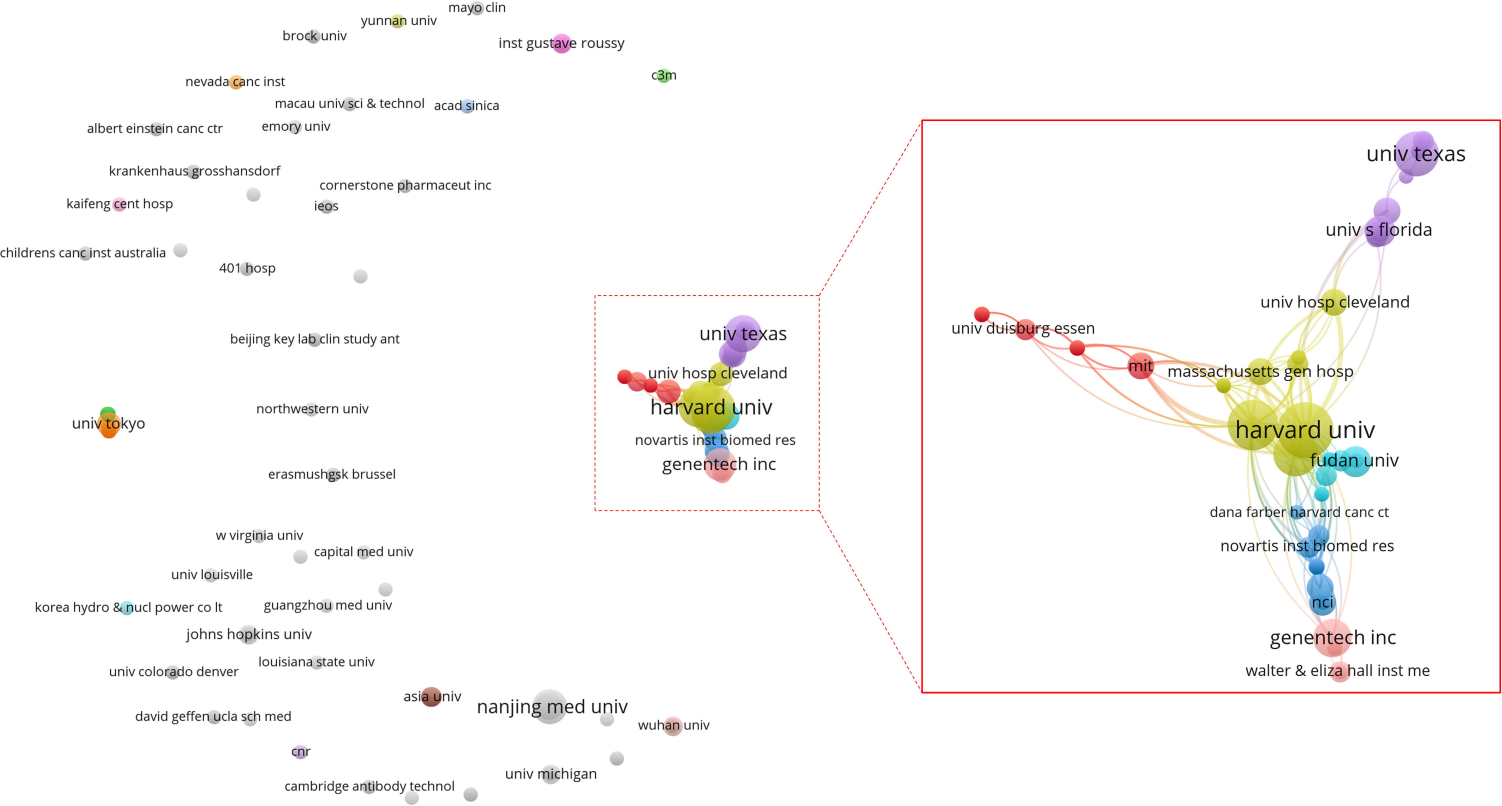
Co-authoring relationship diagram between publishing institutions. Each node symbolizes an institution, with the size of the node corresponding to the volume of articles published by that institution. The connecting lines illustrate co-authorship relationships between institutions, with the line thickness indicating the strength of these collaborative efforts. Additionally, the color of each node signifies the cluster to which it belongs, with each cluster being represented by a distinct color.

A total of 269 institutions contributed to the publication of the high-impact article within this field. Among these, 5 institutions published 15 or more articles (n≥15). The leading three institutions are Harvard University (n=64), Dana-Farber Cancer Institute (n=31) and Genentech (n=24). As illustrated in **Figure 5**, which presents a statistical chart of the top 10 institutions, the United States holds a significant standing in this domain. Apart from a select few leading global institutions, there is minimal variation in the number of publications produced by most institutions. **Figure 6** illustrates a deficiency in collaboration among international institutions. Predominantly, cooperation occurs internally within individual countries, with the United States’ domestic institutions lead in fostering close partnerships in this domain.

### 3.5 Analysis of journals

**Figure 7:**
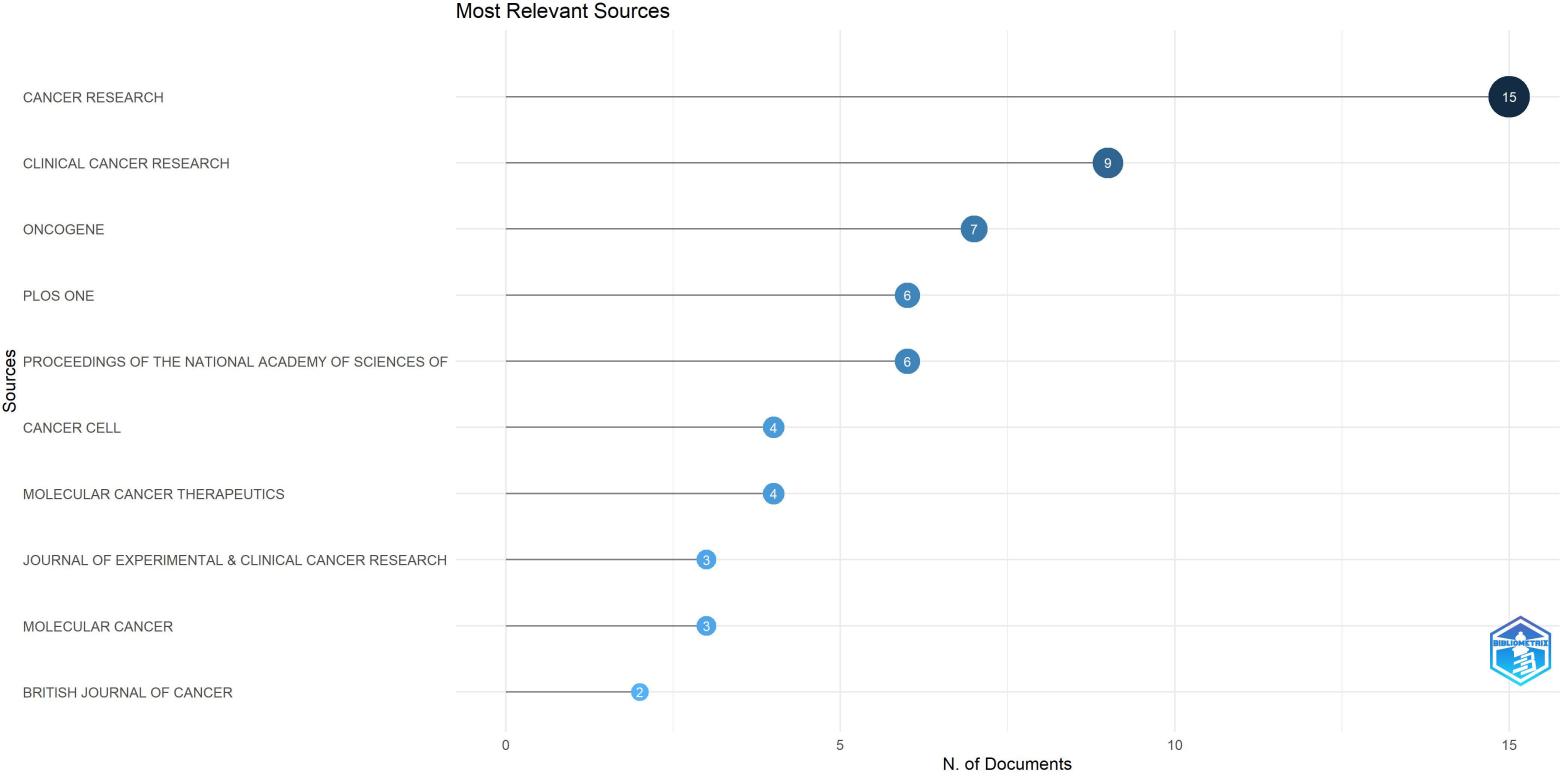
Top 10 journals by the number of articles.

The top 100 highly cited articles retrieved in this study were published across 45 distinct journals, with Impact Factors (IF) ranging from 1.6 to 58.7 as of 2023. Notably, 80% of these journals are classified within the first quartile (Q1) according to the 2023 Journal Citation Reports (JCR) (see **Supplementary Material**). Among these, *CANCER RESEARCH* holds the highest position, contributing 15 articles (see **Figure 7**) and also leading in total citations, with 3,691 citations. However, *CANCER RESEARCH* IF 12.5 in 2023 was not the highest among the 45 journals analyzed. *NATURE MEDICINE*, (n = 2; IF(2023) was 58.7), demonstrated a significant advantage in terms of IF. All journals ranked within the top 10 for publication volume achieved a Q1 classification in the 2023 JCR rankings. The total citations and journal IF of 45 journals were sorted (see **Supplementary Materials** for details), and the top 10 journals were selected respectively (see **Table 2**). Among the top ten journals ranked by total citations, 8 have accrued more than 1,000 citations each. Notably, despite having published only a single article, the journal *SCIENCE* ranks fifth in total citations, underscoring its significant influence. All of the top ten journals possess an IF exceeding 10, indicating their status as leading publications within their respective fields. This suggests that the majority of highly influential articles in this study were published in high-impact journals (refer to **Supplementary Material**).

**Table 2:** The top 10 journals by total citations/journal impact factor.

Rank (Total citations)	Journal	Articles	Total citations	JIF	JCI
1	*CANCER RESEARCH*	15	3,691	12.5	1.99
2	*CLINICAL CANCER RESEARCH*	9	2,403	10.4	2.52
3	*CANCER CELL*	4	1,603	48.8	7.57
4	*ONCOGENE*	7	1,397	6.9	1.47
5	*SCIENCE*	1	1,389	44.8	9.90
6	*PLOS ONE*	6	1,249	2.9	0.88
7	*PROCEEDINGS OF THE NATIONAL ACADEMY OF SCIENCES OF THE UNITED STATES OF AMERICA*	6	1,329	9.4	2.40
8	*NATURE*	2	1,006	50.5	11.30
9	*MOLECULAR CANCER THERAPEUTICS*	4	922	5.4	1.18
10	*NATURE MEDICINE*	2	922	58.7	13.63
Rank (JIF)	Journal	Articles	Total citations	JIF	JCI
1	*NATURE MEDICINE*	2	922	58.7	13.63
2	*NATURE*	2	1,006	50.5	11.30
3	*CANCER CELL*	4	1,603	48.8	7.57
4	*SCIENCE*	1	1,389	44.8	9.90
5	*MOLECULAR CANCER*	3	532	27.7	5.25
6	*JOURNAL OF THORACIC ONCOLOGY*	1	175	21.1	4.29
7	*NATURE CELL BIOLOGY*	1	393	17.3	2.94
8	*CELL DEATH AND DIFFERENTIATION*	1	326	13.7	1.97
9	*JOURNAL OF CLINICAL INVESTIGATION*	1	150	13.3	3.45
10	*CANCER RESEARCH*	15	3,691	12.5	1.99

### 3.6 Analysis of authors

**Figure 8:**
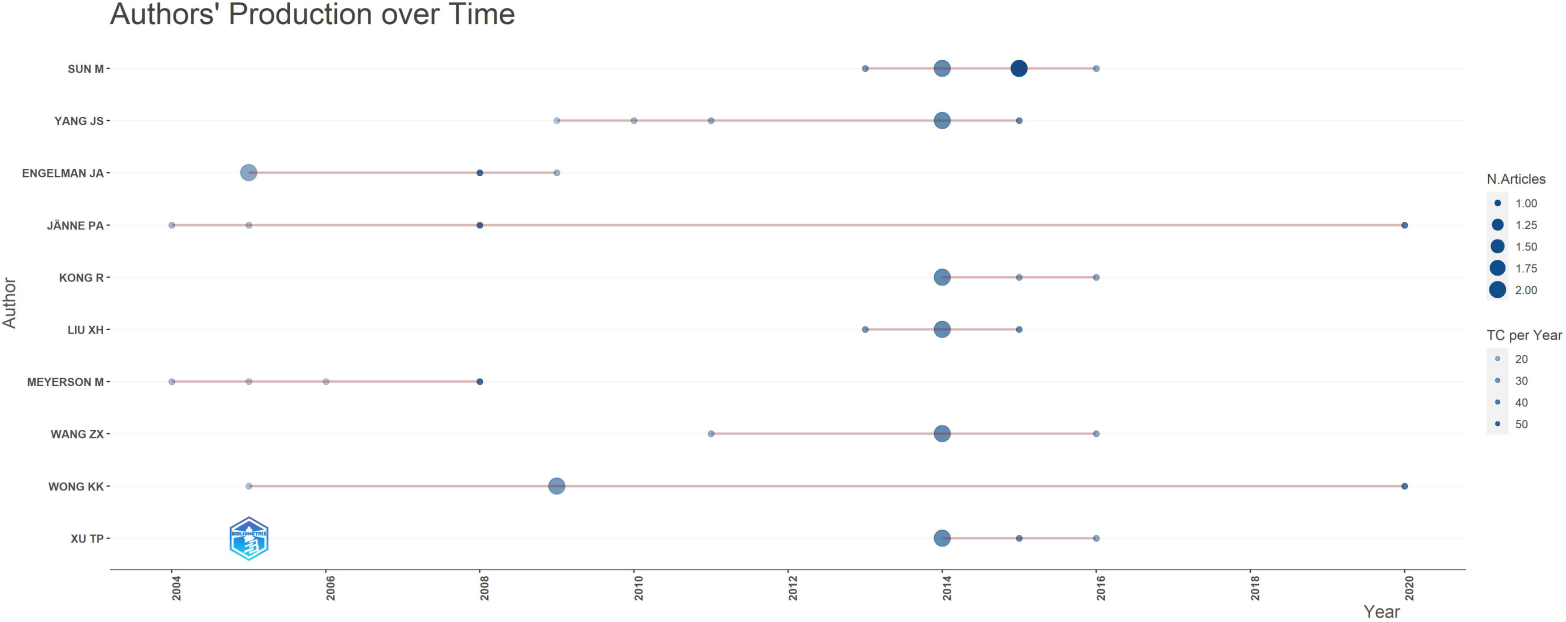
Time distribution of the top 10 authors according to the number of articles.

**Figure 9:**
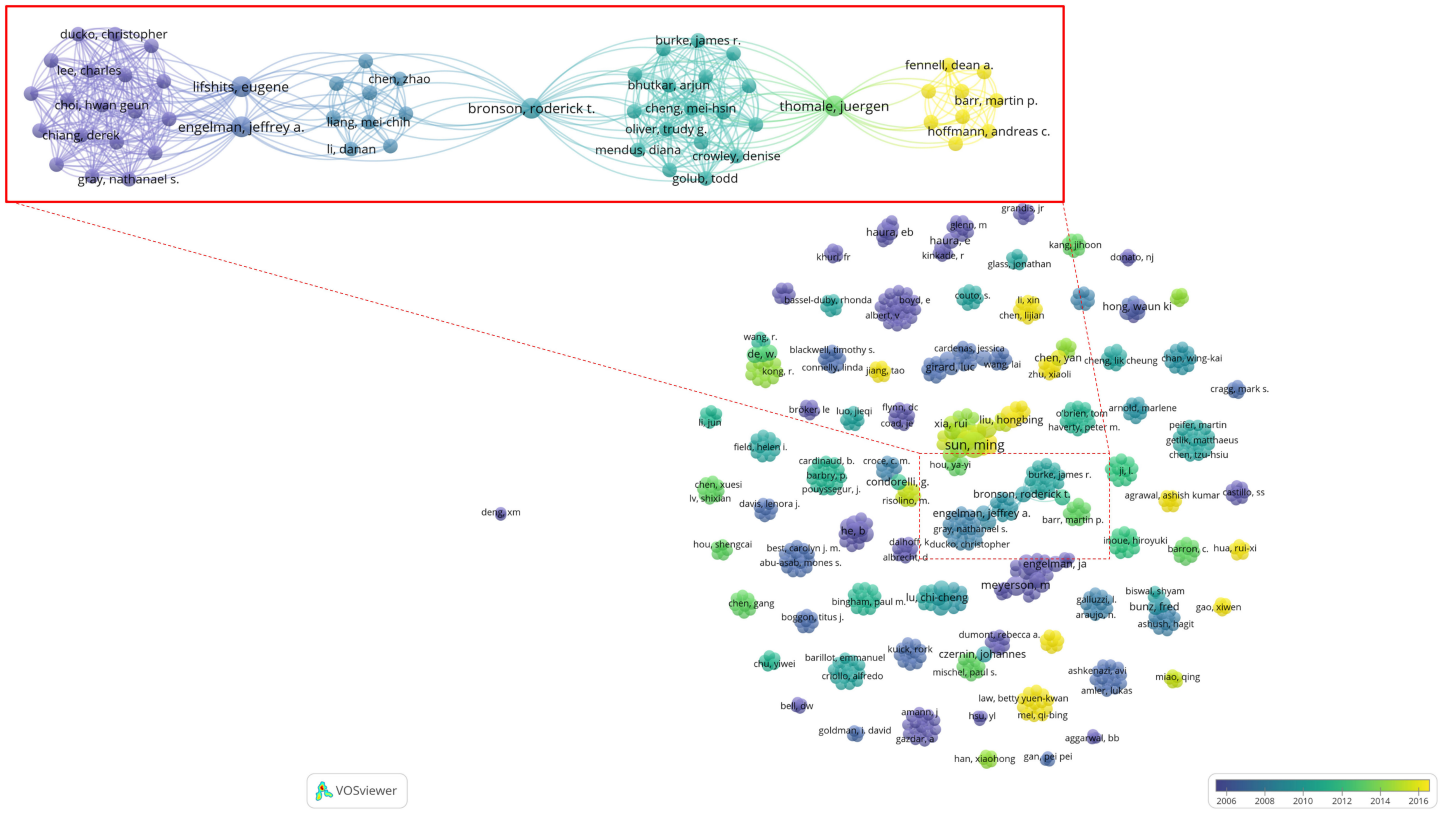
Co-authorship relationship diagram between authors. Each node symbolizes an author, with the size of the node indicating the number of articles published by that author. The connecting lines denote co-authorship relationships, where the thickness of the lines reflects the strength of these collaborations. The color of each node corresponds to the timeline of publication, as depicted in the graph located at the bottom right.

A total of 934 authors participated in the production of high-impact studies within the field. Notably, SUN M and YANG JS emerged as the most prolific authors, each having published 6 articles. However, the works authored by SUN M have garnered a higher total number of citations, as well as a greater average number of citations per year, compared to those authored by YANG JS (refer to **Table 1**). Based on the publication time distribution diagram for the top 10 authors (**Figure 8**), there was a notable concentration of articles published in 2014, with several authors belonging to the same research team. For instance, researchers SUN M, YANG JS, XU TP, LIU XH, KONG R and WANG ZX, affiliated with Nanjing Medical University, have contributed significantly to high-impact studies investigating the pivotal role of non-coding RNA (ncRNA) in the proliferation and apoptosis of NSCLC (26, 30, 39, 49, 64, 66, 79). ENGELMAN JA, WONG KK and MEYERSON M, affiliated with Harvard Medical School, collaborate on research focused on developing effective strategies for the treatment of EGFR-mutated LC (42, 44, 52, 59, 65, 67). As illustrated in **Figure 9**, the collaboration diagram among authors does not constitute a cohesive network. Instead, author collaboration predominantly occurs within individual research teams. Notably, several key research teams have been identified, including BRONSON RT, THOMALE J, LIFSHITS E, ENGELMAN JA and others. The researchers continue to novel strategies to address EGFR mutations in NSCLC. Their findings indicate that targeting heat shock protein 90, along with the simultaneous inhibition of the PI3K-mTOR and MEK signaling pathways may constitute promising therapeutic approaches for these mutations (44, 59). Furthermore, they have identified distinct characteristics of cisplatin-resistant subpopulations within NSCLC cells (72, 102). These scholars, primarily affiliated with prestigious academic institutions, participate in international collaborations to enhance knowledge sharing and collective expertise.

### 3.7 Analysis of keywords

**Figure 10:**
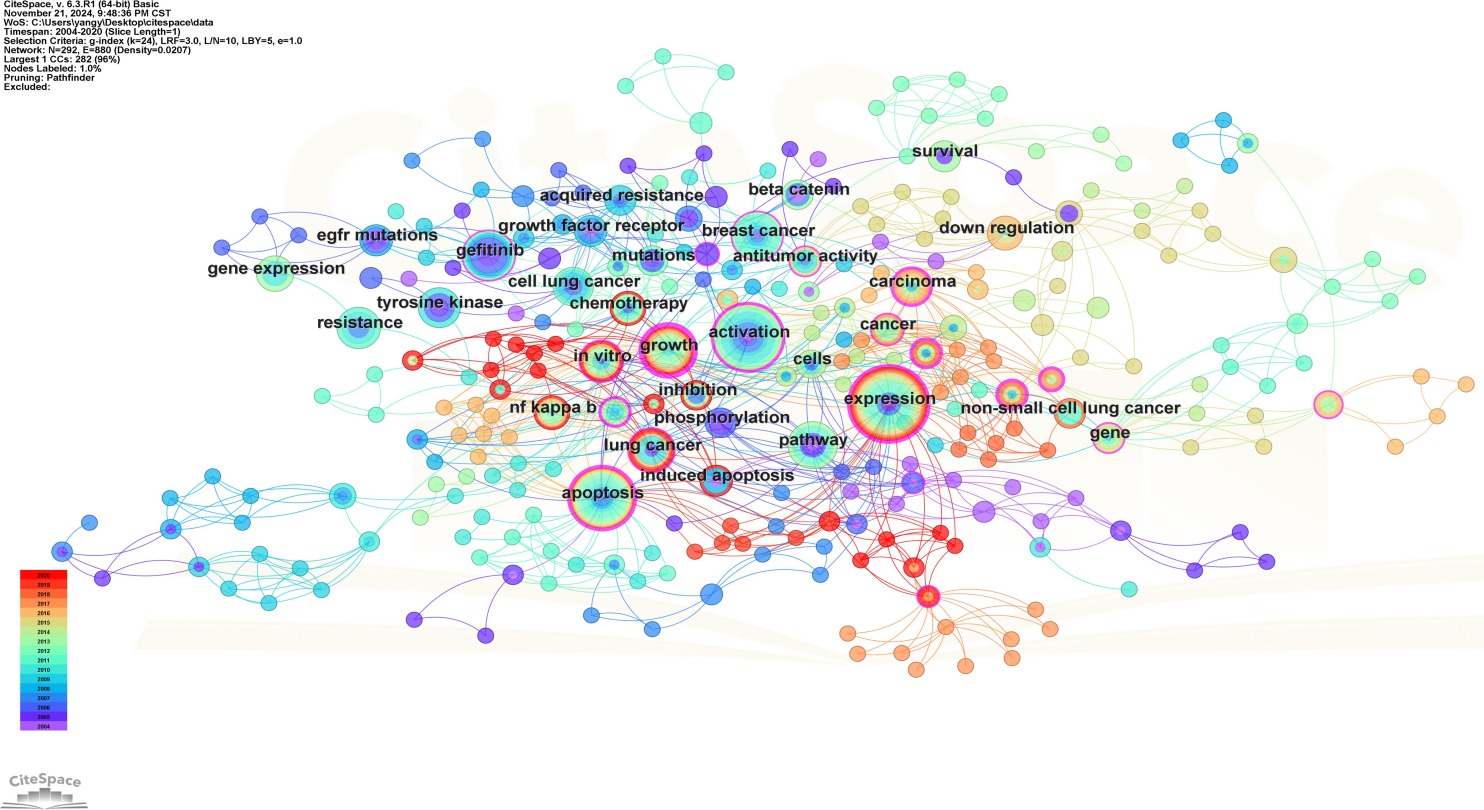
Keywords co-occurrence map. Each node symbolizes a specific keyword, with the size of each node reflecting the frequency of the keyword’s occurrence; a higher frequency results in a larger node. The connecting lines between nodes signify the co-occurrence of keywords. Additionally, the color of each node corresponds to the temporal diagram presented on the left.

There are a total of 539 keywords (including keywords that indicate the relevant meaning) in these 100 articles and there are 7 keywords that appear more than 10 times. Keyword co-occurrence analysis serves as an effective tool for identifying prominent topics within this research domain. **Figure 10** shows the visual network diagram of keyword co-occurrence, in which the keyword “expression” appears most frequently, followed by “activation”, “apoptosis”, “pathway” and “gefitinib”.

**Figure 11:**
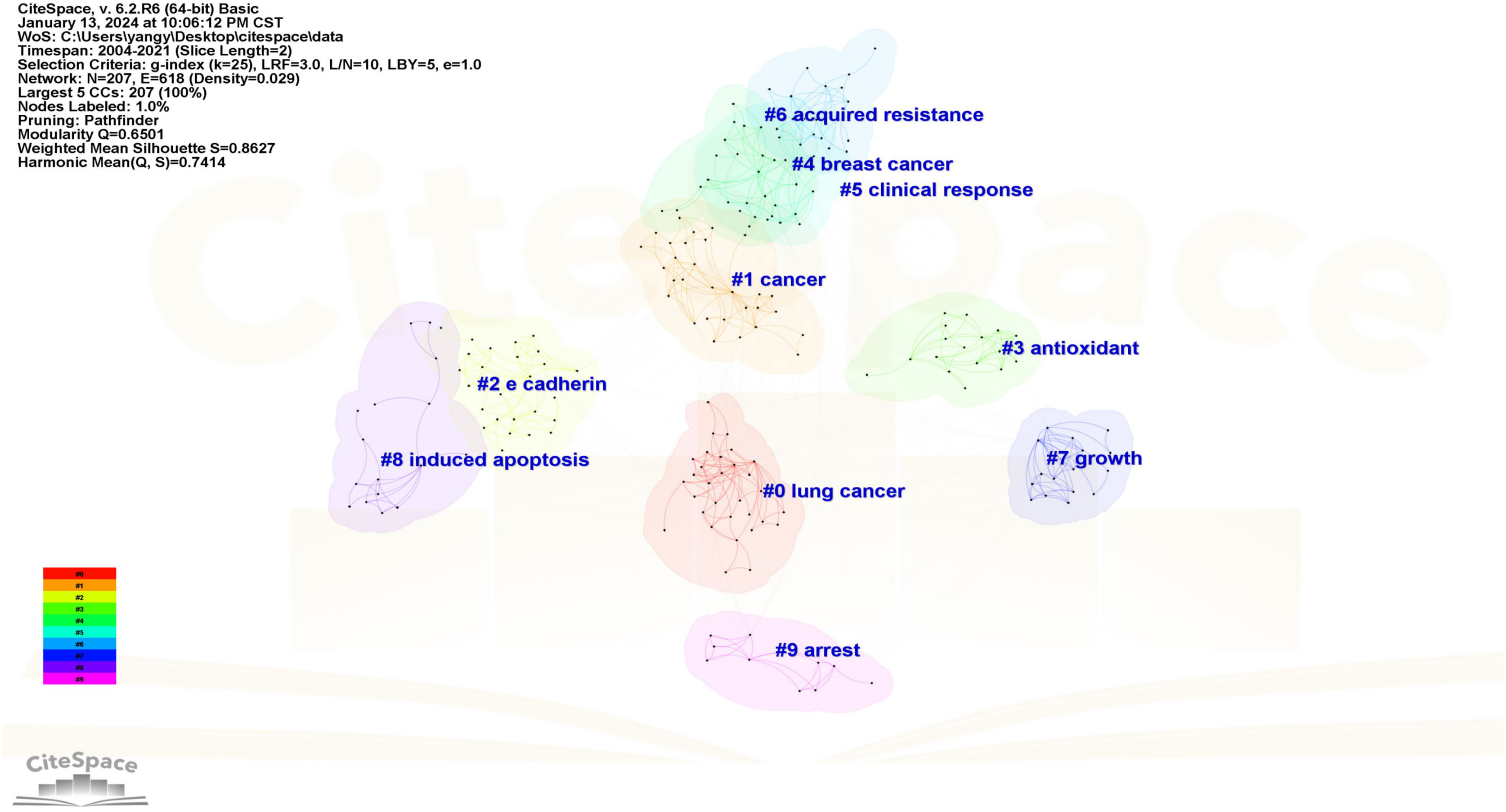
Keywords clustering. Each node symbolizes a specific keyword.

**Figure 12:**
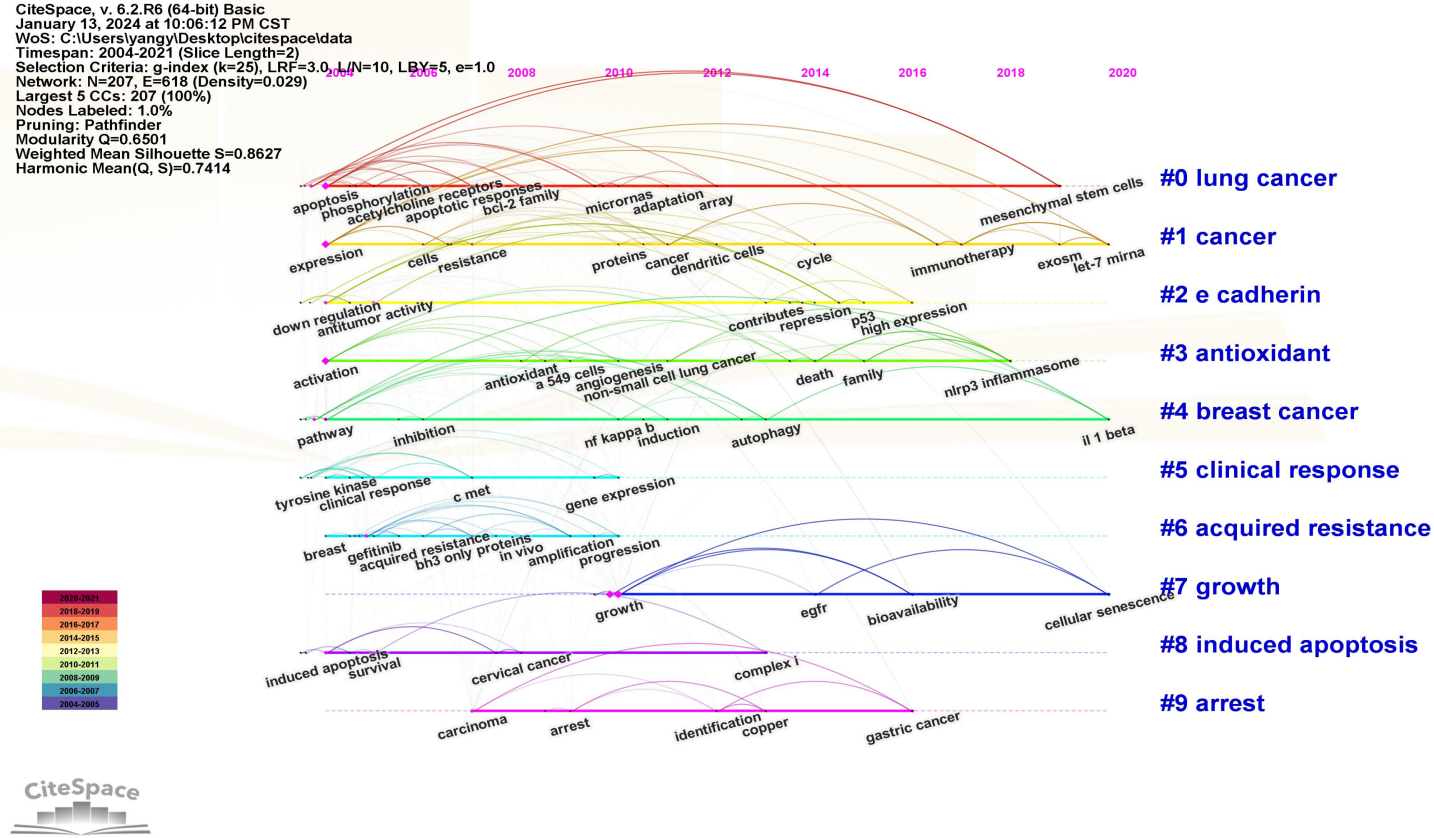
Keywords clustering timeline diagram. The placement of a node along the horizontal axis indicates the initial occurrence of the corresponding keyword. A node positioned further to the right on the horizontal axis signifies a later appearance of the keyword.

The Log-likelihood Rate (LLR) algorithm in Citespace is used for cluster analysis. The analysis results delineated the keywords into 10 distinct clusters, each represented by a unique color. The clustering modularity value (*Q)* is 0.6501 and the average clustering contour value (*S)* is 0.862 7. These metrics suggest that the cluster structure is both significant and reasonable (**Figure 11**). The smaller the cluster label, the more keywords contained in the cluster. So the cluster with the most keywords is clusters #0, which focuses on the study of apoptosis of mitochondrial pathway. Clusters #1, #5, and #6 explore EGFR tyrosine kinase inhibitors (TKI) to inhibit signal transduction and accelerate apoptosis. Clusters #3 focuses on the relationship between antioxidant system and apoptosis. Clusters #2, #4 and #7 focus on the pathways and bioactive substances that promote cell proliferation and anti-apoptosis. Clusters #8 and #9 examine the link between tumor suppressor genes and proteins encoded by tumor suppressor genes and cell apoptosis (see **Supplementary Material**). The keyword clustering time plot (**Figure 12**) shows that “mesenchymal stem cells”, “let-7-miRNA”, “IL-1β” and “cellular senescence” have been prominent keywords recently dicating current research trends and potential future hotspots in this field.

### 3.8 Analysis of references

A total of 3,855 references were referenced in this study, with 15 cited over 5 times by these 100 highly cited articles. The top 10 references in the number of co-citations are listed in **Table 3**. These were analyzed and visualized using VOSviewer, revealing a tightly connected network that underpins and advances the research field (see **Supplementary Material**). The first cited reference is the EGFR mutations in lung cancer: Correlation with clinical response to gefitinib therapy written by PAEZ JG. et al(2004) (114), which has been cited 21 times in these 100 articles, with a total of 7,773 citations. This study reported that EGFR mutations can predict the sensitivity of LC patients to gefitinib. Secondly, Activating mutations in the epidermal growth factor receptor underlying responsiveness of non-small-cell lung cancer to gefitinib, written by LYNCH TJ. et al. (2004) (115), was cited 18 times in the 100 articles, with a total of 9,113 citations. This study sequenced the EGFR gene in 16 LC patients treated with gefitinib, finding gene mutations in 8 of the 9 patients who responded well, but none in the 7 who did not. This is the first evidence linking EGFR mutations to drug efficacy. These two influential articles initiated precise targeted therapy for LC. Later studies (116, 117) revealed that some tumors progressing after EGFR-TKI treatment had mutations at exon 20, site 790 of the EGFR gene, leading to gefitinib resistance. The ISEL study (118) found that gefitinib did not significantly improve overall survival in the general or adenocarcinoma populations, though non-smokers and Asian patients may benefit. However, at the same time, the large phase III study BR21 (119) demonstrated that erlotinib extends survival and alleviates symptoms in previously treated NSCLC patients compared to placebo, which further confirmed the status of EGFR-TKI. A phase 3 open-label study (IPASS) (120) found gefitinib to be more effective than carboplatin plus paclitaxel for initially treating lung adenocarcinoma in East Asian non-smokers or former mild smokers, suggesting that patients with EGFR mutations benefit most from gefitinib as a first-line treatment. It can be seen that in a series of multi-center clinical studies carried out from 2003 to 2005 showed expanding clinical benefits and increased significance of gefitinib for patients with EGFR mutation, making it a major research focus.

**Table 3:** The top 10 reference ranked by the co-citation.

Rank	Title	Journal	First author	Year	Co-citation	Total citation (WoS Core)
1	EGFR mutations in lung cancer: Correlation with clinical response to gefitinib therapy	*SCIENCE*	PAEZ JG	2004	21	7,773
2	Activating mutations in the epidermal growth factor receptor underlying responsiveness of non-small-cell lung cancer to gefitinib	*NEW ENGLAND JOURNAL OF MEDICINE*	LYNCH TJ	2004	18	9,113
3	Aberrant epidermal growth factor receptor signaling and enhanced sensitivity to EGFR inhibitors in lung cancer	*CANCER RESEARCH*	AMANN J	2005	10	372
4	EGF receptor gene mutations are common in lung cancers from “never smokers” and are associated with sensitivity of tumors to gefitinib and erlotinib	*PROCEEDINGS OF THE NATIONAL ACADEMY OF SCIENCES OF THE UNITED STATES OF AMERICA*	PAO W	2004	10	3,570
5	Gefitinib induces apoptosis in the EGFRL858R non-small-cell lung cancer cell line H3255	*CANCER RESEARCH*	TRACY S	2004	10	309
6	Akt/protein kinase B is constitutively active in non-small cell lung cancer cells and promotes cellular survival and resistance to chemotherapy and radiation	*CANCER RESEARCH*	BROGNARD J	2001	8	835
7	EGFR mutation and resistance of non-small-cell lung cancer to gefitinib	*NEW ENGLAND JOURNAL OF MEDICINE*	KOBAYASHI S	2005	8	3,286
8	Efficacy of gefitinib, an inhibitor of the epidermal growth factor receptor tyrosine kinase, in symptomatic patients with non-small cell lung cancer - A randomized trial	*JAMA-JOURNAL OF THE AMERICAN MEDICAL ASSOCIATION*	KRIS MG	2003	8	2,158
9	Gefitinib-sensitizing EGFR mutations in lung cancer activate anti-apoptotic pathways	*SCIENCE*	SORDELLA R	2004	8	1,391
10	Erlotinib in previously treated non-small-cell lung cancer	*NEW ENGLAND JOURNAL OF MEDICINE*	SHEPHERD FA	2005	7	4,566

### 3.9 Analysis of research hotspots

Given that the articles included in this study do not encompass articles from 2021 onwards, in order to understand the research hotspots in this field in recent years, the top 10 most-cited articles published between 2021 and March 8, 2024 were retrieved. Using the same retrieval method described previously (see **Supplementary Materials** for specific articles). Subsequently, the authors’ keywords were compiled, and synonymous keywords were consolidated to quantify their frequency of occurrence. Considering the top ranking “non-small cell lung cancer” and “apoptosis” as thematic keywords in this study, these terms were excluded from further analysis. Among the remaining keywords, “circular RNA”, “Nrf2”, “autophagy” and “ferroptosis” emerged as the most frequently occurring, each appearing twice. This keyword analysis suggests that recent research in this field may focus on promoting apoptosis in NSCLC by regulating circular RNA (circRNA) and targeting the nuclear factor erythroid 2-related factor 2 (Nrf2) signaling pathway.

## 4 Discussion

This study seeks to identify the top 100 most-cited articles pertaining to the apoptosis of NSCLC over past two decades and to analyze their bibliometric characteristics. The volume of research on NSCLC apoptosis has shown a consistent upward trend in recent years, reflecting the growing scholarly interest in this area. To investigate the current state of research and emerging focal points within this domain, this study compiles and examines the most frequently cited articles from the past twenty years, thereby offering a valuable reference for future research endeavors.

LC ranks as the second most prevalent malignancy globally, with approximately 1,796,144 fatalities reported in 2020. The overall 5-year survival rate for LC patients ranges from 10% to 20% following diagnosis (1). However, for those diagnosed at stage IV, the 5-year survival rate diminishes to a mere 5%. Notably, the majority of patients are diagnosed at an advanced stage of the disease (121). The therapeutic approaches for NSCLC typically encompass surgical intervention, chemotherapy, radiotherapy, molecular targeted therapy, immunotherapy, among others (122). However, the emergence of personalized treatment strategies for NSCLC has notably enhanced patient prognosis, particularly through the advancements in molecular targeted therapy and immunotherapy (123). Recent studies indicate that the mortality rate of NSCLC has declined more significantly than its incidence rate in recent years, coinciding with the approval of targeted therapies (124). Currently, numerous targets and targetable pathways have been identified, including EGFR, ALK, RET, MET, PI3K/AKT/mTOR, RAS-MAPK, and NTRK/ROS1, among others (125, 126).

In this study, while the annual number of studies on apoptosis in NSCLC has consistently increased, the number of highly cited articles exhibits a fluctuating pattern and has demonstrated a negative growth trend over the past two decades. Upon reviewing the titles and abstracts of the 100 highly cited articles included in this analysis, they can be categorized into two main categories that articles are nearly equivalent in quantity. The first area of focus pertains to the mechanisms of apoptosis induced by pharmacological agents and compounds, as well as the mechanisms underlying drug resistance. Specifically, 17 articles address targeted therapies, primarily those involving EGFR-TKIs; 16 articles explore the effects of chemical compounds, including nicotine and curcumin; and 6 articles examine the role of chemotherapeutic agents. The other category pertains to the relationship between the expression of genes, proteins and other biomarkers and the process of apoptosis. Specifically, 17 articles focus on the involvement of related proteins in mediating apoptosis, 11 articles examine the direct or indirect role of microRNAs in regulating multiple apoptotic pathways, and 7 articles explore the regulation of apoptosis by long non-coding RNAs (lncRNA). The United States and China are identified as the first and second leading countries (regions) in terms of published articles (**Table 1**). The United States holds the distinction of having the highest number of published articles, with a total of 60. Between 2004 and 2007, the United States contributed to over 80% of published articles. However, post-2008, there was a noticeable decline in the proportion of articles originating from the United States. The proportion of published articles originating from China has shown a continuous increase since 2013. Since that time, the annual number of highly cited articles from China has surpassed those from other countries. This trend underscores the significant contributions made by both the United States and China in this field. Regarding publishing institutions, the United States maintains a dominant position, with seven of the top 10 most productive institutions being based there, highlighting the critical role of the United States in this area of research.

The 100 articles analyzed were published in 45 distinct journals. Although none of these articles appeared in the top four medical journals, over 60% were published in journals with a high IF and were published in journals classified as JCR Q1, indicating that the field of NSCLC apoptosis research is well-established and has garnered significant attention from the academic community. Notably, some studies have been featured in prestigious scientific journals such as *NATURE* and *SCIENCE*. Additionally, publications have appeared in leading cell biology journals, including *NATURE MEDICINE*, *CANCER CELL*, *NATURE CELL BIOLOGY*, *CELL DEATH AND DIFFERENTIATION*. In this study, it was observed that the majority of articles with a higher number of citations were published in journals possessing a higher IF. However, journals with a greater number of co-citations did not necessarily exhibit a higher IF. Among the 45 journals analyzed, the lowest IF recorded was 1.6, demonstrating that even journals with a lower IF can produce highly cited articles. Furthermore, previous research has indicated that there is no statistically significant correlation between high citations counts and a journal’s IF (127). This finding underscores the importance of prioritizing the quality of individual papers alongside the journal IF.

Highly cited articles frequently represent the research focal points within a specific field, with keywords serving as significant indicators (128). Among the 100 articles analyzed, the most frequently occurring keyword is “expression”, followed by “activation”, “apoptosis”, “pathway and “gefitinib”. In the research articles examined, apoptosis is primarily induced through the regulation of the expression of microRNAs, tumor suppressors, EGFR, cell cycle proteins, and anti-apoptotic proteins, among others. Simultaneously, the study examined the activation of signaling pathways, transcription factors and various proteases involved in the regulation of apoptosis. The primary focus was on the activation of diverse signaling pathways and their impact on apoptosis. For instance, the activation of pathways such as PI3K/AKT, Wnt/β-catenin, and NF-κB by drugs or compounds plays a crucial role in modulating apoptosis in NSCLC. The findings indicate that the apoptosis pathway and the mechanisms by which drugs induce apoptosis remain among the most prominent topics of interest in the field.

Research priorities are subject to change over time. Consequently, a further examination and analysis of the top 10 most highly cited articles post-2021 revealed that the predominant keywords include “circular RNA”, “Nrf2”, “autophagy” and “ferroptosis”. This suggests that the regulation of circRNA and the targeting of the Nrf2 signaling pathway to induce apoptosis in NSCLC may constitute a significant focus of contemporary research. The dysregulated expression and control of circRNA are recognized as playing a crucial regulatory role in NSCLC (129). Two of the studies included in the analysis have demonstrated that silencing tumor-derived exosomal circRNA_102481 can enhance apoptosis in EGFR-TKIs resistant NSCLC, while the loss of circRNA vacuolar membrane protein 1 similarly promotes apoptosis of cisplatin-resistant NSCLC (129, 130). Additionally, one of the articles reported that kaempferol effectively induces down-regulation of Nrf2 mRNA and disrupts Nrf2 downstream signaling in a redox-independent manner, thereby facilitating apoptosis in NSCLC (131). Four articles have concurrently examined ferroptosis and apoptosis, as well as the interplay between autophagy and apoptosis, thereby analyzing the relationship and interactions between the processes. This research has significantly enhanced the understanding of cell death mechanisms (132–135).

The principal mechanisms of cancer cell death encompass apoptosis, pyroptosis, autophagy and necrosis, with apoptosis being particularly significant (136). Apoptosis is characterized as a gene-regulated, programmed cell death process that maintains cellular homeostasis without eliciting an inflammatory response. It can be initiated via an extrinsic pathway, triggered by cell surface death receptors, or an intrinsic pathway, primarily activated by mitochondrial damage. Both pathways predominantly involve the mediation of effector caspases (137–139). The canonical mechanism of apoptosis involves the activation of the caspase pathway. Initiator caspases, including caspase-2, -8, and -9, play a critical role in triggering the apoptotic cascade by activating downstream effector caspases, such as caspase-3, -6, and -7, which ultimately execute the apoptotic process (140–142). The exogenous apoptotic pathway is activated by various death receptor stimuli, including Fas, tumor necrosis factor (TNF) receptors, and the TNF-related apoptosis-inducing ligand receptor (TRAILR), among others. These membrane receptors, which participate in the apoptotic process, are members of the TNF receptor superfamily. Within this family, TNF and Fas serve as the primary ligands that activate these receptors, thereby initiating the TNF signaling pathway (143, 144). Endogenous apoptotic pathways are activated by factors such as DNA damage, energy deprivation, and hypoxia, leading to the dephosphorylation and cleavage of pro-apoptotic proteins, their translocation to the mitochondria, and the subsequent induction of apoptosis (143). Additionally, the endoplasmic reticulum (ER) plays a critical role in apoptosis. Dysfunction in protein folding within the ER results in the accumulation of unfolded or misfolded proteins in its lumen, thereby inducing ER stress and contributing to the apoptotic process (145).

Numerous signaling pathways are implicated in the regulation of apoptosis in NSCLC, including but not limited to STAT3, Wnt/β-catenin, NF-κB, PI3K/AKT, mTOR, MAPK/Slug, ROS, p53, and Nrf2. These pathways can modulate apoptosis through both endogenous and exogenous mechanisms (146). Additionally, a range of pro-apoptotic regulatory factors are involved this process, including cytochrome C, Smac/DIABLO, Omi/HtrA2, AIF, and endonuclease G, among others (147). Numerous genes and proteins play crucial roles in the mechanism of apoptosis. For instance, TP53 is a significant tumor suppressor gene that can arrest the cell cycle and promote apoptosis. P53, a widely studied pro-apoptotic transcription factor, is a key participant in the cell cycle regulation. Apoptosis signal induced by cancer cells is p53-dependent (148, 149). For instance, the BCL-2 protein family plays a crucial role in the regulation of apoptosis and is categorized into three distinct subgroups: anti-apoptotic proteins (including BCL-2, BCL-xL, BCL-w, MCL-1, and BFL-1), multi-BH domain pro-apoptotic proteins (such as BAK, BAX, and BOK), and BH3-domain only pro-apoptotic proteins (such as BIM, BAD, BID, NOXA, BIK, HRK, BMF and PUMA) (150, 151). Venetoclax, the sole globally approved Bcl-2 selective inhibitor, has received approval for the treatment of relapsed or refractory chronic lymphocytic leukemia and acute myeloid leukemia in elderly patients. However, its clinical indications for other diseases remain under investigation (152).

Consequently, targeting the apoptotic pathway represents a potent strategy for the treatment of NSCLC. Anticancer agents exert their effects by inducing apoptosis and arresting the cell cycle (153). These agents facilitate apoptosis by inhibiting the expression of anti-apoptotic proteins, upregulating pro-apoptotic proteins, decreasing mitochondrial membrane potential, and promoting the release of cytochrome C (154, 155). Mutations in EGFR, human epidermal growth factor receptor-2 (HER-2), or Kirsten rat sarcoma virus oncogene homolog (KRAS) result in increased tumor cell proliferation and diminished apoptosis (156). A critical mechanism by which EGFR-TKIs exert therapeutic effects in EGFR-mutated NSCLC is through the induction of apoptosis. This process is mediated by the regulation of BCL-2 family protein expression, with the pro-apoptotic protein BIM plays a pivotal role (157, 158). Nevertheless, drug resistance in patients with NSCLC patients with NSCLC remains a significant challenge. Drug resistance can be categorized into primary and acquired types. Primary drug resistance is present in a minor subset of cancer cells that have not previously undergone treatment, whereas acquired drug resistance frequently proves difficult to circumvent (159). The mechanisms underlying drug resistance in LC are predominantly associated with gene mutations, gene deletions, gene amplifications, pharmacokinetics factors, targeting of oncogenes, and drug-induced apoptosis (160, 161). Inhibition of apoptosis represents a principal factor contributing to multidrug resistance in tumors, with the PI3K/AKT signaling pathway playing a crucial role in the regulation of this resistance in cancer (162). Residual cancer cells that persist following drug treatment can act as reservoirs for acquired drug resistance. Therefore, augmenting initial drug-induced apoptosis and minimizing residual lesions is a promising strategy to mitigate drug resistance (65). Currently, research on EGFR-TKI has progressed to the fourth generation, with compounds such as EAI045, OBX02-011, LS-106 and CH7233163 under investigation. These fourth-generation drugs are anticipated to address multiple mechanisms of drug resistance, including those associated with third-generation inhibitors, which are predominantly exemplified by resistance to osimertinib (163). Inhibition of apoptosis has implications for tumor drug resistance. In the context of hematological malignancies, the use of BCL-2 family inhibitors in combination therapies has been shown to effectively enhance apoptosis and diminish drug resistance (164, 165). A Phase I clinical trial investigating the combination of osimertinib and ABT-263 (Navitoclax) for the treatment of LC patients with EGFR mutations, following the failure of EGFR-TKI therapy, utilized a dosage regimen of 80 mg/day of osimertinib and 150 mg/day of Navitoclax. The treatment was both efficacious and well-tolerated by the patient cohort (164). A study has demonstrated that the upregulation of BCL2L1 expression constitutes the primary alteration observed in cells that develop resistance to EGFR-TKI (166).

LncRNAs constitute a significant focus within the literature reviewed in this study. These molecules are intricately associated with the pathogenesis of NSCLC and are critical in modulating drug resistance mechanisms. Evidence indicates that lncRNAs contribute to the development of cisplatin resistance in LC, potentially influencing resistance to taxanes and acquired resistance to EGFR-TKIs (167). The lncRNA/PTEN axis is pivotal in modulating the sensitivity of LC cells to chemotherapeutic agents. Research indicates that miRNA-221 contributes to the development of cisplatin resistance in LC by down-regulating PTEN (168). The suppression of LINC01296 has been shown to significantly enhance the sensitivity of NSCLC to paclitaxel (169). LncRNAs play a crucial role in the emergence and progression of resistance to EGFR-TKIs in NSCLC by modulating various molecular pathways. Certain lncRNAs enhance the expression of oncogenes, thereby facilitating tumor cell proliferation, survival, and resistance to EGFR-TKI. Additionally, some lncRNAs suppress the expression of tumor suppressor genes, induce the epithelial-mesenchymal transition (EMT) process, and ultimately contribute to EGFR-TKI resistance. This resistance is partially mediated through the activation of the Rho-associated protein kinase (ROCK) and mesenchymal-epithelial transition factor (MET) signaling pathways. Certain lncRNAs suppress the expression of tumor suppressor genes and induce the EMT process, ultimately contributing to resistance against EGFR-TKIs. This resistance is partially mediated through the activation of the ROCK and MET signaling pathways (170). CircRNAs represent a novel category of endogenous ncRNAs characterized by a covalently closed-loop structure. Currently, substantial evidence suggests that circRNAs are capable of modulating gene expression across various levels. These regulatory functions include influencing the transcription of their parental genes, affecting the splicing processes of their linear counterparts, serving as microRNA sponges, modulating the activity of RNA-binding proteins, and facilitating the encoding of peptides (171). Aberrant circRNAs play a significant role in various developmental processes of malignant tumors, such as tumorigenesis, growth, metastasis, apoptosis, angiogenesis, and resistance to chemotherapy (172). CircRNAs have been identified as crucial elements within the intricate regulatory network of the KRAS pathway across different cancer types. Investigating the involvement of circRNAs in the KRAS pathway could enhance strategies for cancer detection and treatment (173). Currently, numerous studies have identified that certain circRNAs are down-regulated in tumors, potentially contributing to the inhibition of tumor chemotherapy resistance. Conversely, their up-regulation may enhance chemotherapy resistance (174). These circRNAs can modulate the resistance of various tumors to conventional chemotherapy or immunotherapy, either positively or negatively (172). Consequently, circRNAs hold promise as potential diagnostic, prognostic, and therapeutic targets for cancer. Furthermore, they offer a promising avenue for research aimed at addressing the challenge of drug resistance in LC. Nrf2 is a pivotal transcription factor that plays an essential role in the oxidative stress response. It possesses the capacity to modulate apoptosis in human cancer cells, exhibiting a dualistic function. The interaction between Nrf2 and apoptosis processes can significantly influence cancer progression (175). The accumulation of Nrf2 within cancer cells, along with its cytoprotective effects, can be attributed to somatic mutations in Nrf2, Keap1, or CUL3. Additionally, the down-regulation of Keap1, as well as the interactions of p62/Sqstm1 and p21 with Keap1, can impact Nrf2 levels (176). Mutations within the Keap1/Nrf2 signaling pathway have been implicated in the development of resistance to cancer chemotherapy, targeted therapies, and radiotherapy (177). Yoon Jin Lee. et al. (2020) (178) demonstrated that Nrf2 can enhance the survival rate of cancer cells by inhibiting apoptosis. The overexpression of Nrf2 may be associated with tumorigenesis and tumor progression. In cancer cells, the overexpression of NRF2, resulting from constitutive activation or hyperactivation, facilitates their survival in environments rich in ROS, which is closely associated with enhanced proliferation and increased resistance to chemotherapy (179). Currently, research on NRF2 activators is extensive, whereas investigations into NRF2 inhibitors remain limited (180). NRF2 represents a promising therapeutic target or a potential avenue for targeted therapy, given the frequent occurrence of molecular pathway disruptions or loss-of-function mutations in tumors.

## 5 Conclusion

According to the search, no bibliometric study on the same topic as this study was found. This study utilized the WOSCC, recognized as a relatively authoritative citation database (181). Through an analysis of the 100 most highly cited articles, the study identified the most influential countries (regions), institutions, journals and authors in the field of NSCLC apoptosis. Using bibliometric methods, the research summarized the current status and key areas of interest within this field and projected future research trends based on the temporal distribution of keywords.

Apoptosis-based therapeutic strategies have undergone extensive development over the years. Currently, significant advancements have been achieved in the formulation of novel pharmacological agents and in addressing the challenge of existing drug resistance. Despite the absence of approved anti-apoptotic drugs specifically for NSCLC, these therapies hold considerable potential for future applications. The resistance to anti-tumor drugs remains a formidable challenge within oncology, with the suppression of apoptosis identified as a principal factor contributing to the multidrug resistance observed in tumors. LncRNAs, circRNAs and Nrf2 represent promising therapeutic targets and strategies for addressing cancer drug resistance. The advancement of drugs targeting these molecules holds significant clinical value. Nonetheless, the development of Nrf2 inhibitors aimed at overcoming tumor multidrug resistance remains in its nascent stages, with limited research conducted on these inhibitors to date. NcRNAs have been demonstrated to play a critical role in the development of drug resistance across various therapeutic modalities, including chemotherapy, targeted therapy, immunotherapy, and others, with particular emphasis on lncRNAs and circRNAs. Modulating dysregulated ncRNAs presents a promising therapeutic strategy to counteract drug resistance. Continued investigation into their regulatory mechanisms in anti-tumor drug resistance is essential for advancing the treatment of NSCLC. Consequently, it is anticipated that this area will become a focal point of future research endeavors. Despite these advancements, significant challenges remain in the development of novel pharmacological agents and in overcoming resistance to existing treatments. Consequently, additional research is unequivocally warranted to advance future developments. It is anticipated that such efforts will yield significant breakthroughs in the diagnosis, treatment, and mitigation of drug resistance in cancer.

## 6 Limitations

Nevertheless, this study is subject to certain limitations: (1) The analysis was conducted using the WOSCC, yet numerous other databases, such as PubMed and Scopus, exist. The citation counts of articles vary across these databases (182), potentially affecting the study’s analytical outcomes based on the database selection. (2) Articles of significant influence may not have accrued sufficient citations to be included in the top 100 due to their recent publication, potentially omitting emerging research trends from the study. (3) The citation count of an article can be influenced by various factors, including the prestige of the publishing journal and the incidence of self-citation, among others. Consequently, while citation count serves as a significant metric for assessing an article’s impact, it should not be considered the sole indicator. (4) Furthermore, this study exclusively included English-language articles from the WOSCC, which may result in the exclusion of potentially influential articles published in other languages.

## Data Availability

The original contributions presented in the study are included in the article/**Supplementary Material**. Further inquiries can be directed to the corresponding authors.
