# Supplementary material for: Top 100 most-cited articles on apoptosis of non-small cell lung cancer over the past two decades: a bibliometrics analysis
Source: Front Immunol. 2025 Jan 13;15:1512349. doi: 10.3389/fimmu.2024.1512349 (PMC11770037; doi:10.3389/fimmu.2024.1512349)
Supplement: Supplementary file 1 [file DataSheet1.docx]

***Supplementary Materials***

**Table S1**. List of the top 100 most cited apoptosis studies in NSCLC.

| Rank | Title | Journal | First author | Year | Total citations  (WoS Core) | Citations/Year  (Rank) |
| --- | --- | --- | --- | --- | --- | --- |
| 1 | Gefitinib-Sensitizing EGFR Mutations in Lung Cancer Activate Anti-Apoptotic Pathways | *SCIENCE* | Sordella, R | 2004 | 1,389 | 66.14(1) |
| 2 | p53-Mediated Activation of miRNA34 Candidate Tumor-Suppressor Genes | *CURRENT BIOLOGY* | Bommer, GT | 2007 | 910 | 50.56(2) |
| 3 | EML4-ALK Fusion Gene and Efficacy of an ALK Kinase Inhibitor in Lung Cancer | *CLINICAL CANCER RESEARCH* | Koivunen, JP | 2008 | 781 | 45.94(3) |
| 4 | Sensitivity to antitubulin chemotherapeutics is regulated by MCL1 and FBW7 | *NATURE* | Wertz, IE | 2011 | 618 | 44.14(4) |
| 5 | Autocrine TNFα Signaling Renders Human Cancer Cells Susceptible to Smac-Mimetic-Induced Apoptosis | *CANCER CELL* | Petersen, SL | 2007 | 483 | 26.83(18) |
| 6 | Modulation of K-Ras-Dependent Lung Tumorigenesis by MicroRNA-21 | *CANCER CELL* | Hatley, ME | 2010 | 481 | 32.07(13) |
| 7 | Death-receptor O-glycosylation controls tumor-cell sensitivity to the proapoptotic ligand Apo2L/TRAIL | *NATURE MEDICINE* | Wagner, KW | 2007 | 475 | 26.39(19) |
| 8 | A common BIM deletion polymorphism mediates intrinsic resistance and inferior responses to tyrosine kinase inhibitors in cancer | *NATURE MEDICINE* | Ng, KP | 2012 | 447 | 34.38(10) |
| 9 | LKB1 Inactivation Dictates Therapeutic Response of Non-Small Cell Lung Cancer to the Metabolism Drug Phenformin | *CANCER CELL* | Shackelford, DB | 2013 | 434 | 36.17(6) |
| 10 | BIM Mediates EGFR Tyrosine Kinase Inhibitor-Induced Apoptosis in Lung Cancers with Oncogenic EGFR Mutations | *PLOS MEDICINE* | Costa, DB | 2007 | 409 | 22.72(25) |
| 11 | p53 controls cancer cell invasion by inducing the MDM2-mediated degradation of Slug | *NATURE CELL BIOLOGY* | Wang, SP | 2009 | 393 | 24.56(20) |
| 12 | Synthetic lethal screen identification of chemosensitizer loci in cancer cells | *NATURE* | Whitehurst, AW | 2007 | 388 | 21.56(27) |
| 13 | Aberrant Epidermal Growth Factor Receptor Signaling and Enhanced Sensitivity to EGFR Inhibitors in Lung Cancer | *CANCER RESEARCH* | Amann, J | 2005 | 372 | 18.60(32) |
| 14 | Long non-coding RNA MEG3 inhibits NSCLC cells proliferation and induces apoptosis by affecting p53 expression | *BMC CANCER* | Lu, KH | 2013 | 358 | 29.83(15) |
| 15 | Non-Small Cell Lung Cancer Cells Expressing CD44 Are Enriched for Stem Cell-Like Properties | *PLOS ONE* | Leung, ELH | 2010 | 351 | 23.40(22) |
| 16 | Potent and selective small-molecule MCL-1 inhibitors demonstrate on-target cancer cell killing activity as single agents and in combination with ABT-263 (navitoclax) | *CELL DEATH & DISEASE* | Leverson, JD | 2015 | 344 | 34.40(9) |
| 17 | Long Noncoding RNA ANRIL Promotes Non–Small Cell Lung Cancer Cell Proliferation and Inhibits Apoptosis by Silencing KLF2 and P21 Expression | *MOLECULAR CANCER THERAPEUTICS* | Nie, FQ | 2015 | 340 | 34.00(11) |
| 18 | miR-210 is overexpressed in late stages of lung cancer and mediates mitochondrial alterations associated with modulation of HIF-1 activity | *CELL DEATH AND DIFFERENTIATION* | Puisségur, MP | 2011 | 326 | 23.29(23) |
| 19 | miR-15a and miR-16 Are Implicated in Cell Cycle Regulation in a Rb-Dependent Manner and Are Frequently Deleted or Down-regulated in Non–Small Cell Lung Cancer | *CANCER RESEARCH* | Bandi, N | 2009 | 318 | 19.88(28) |
| 20 | Gefitinib Induces Apoptosis in the EGFRL858R Non–Small-Cell Lung Cancer Cell Line H3255 | *CANCER RESEARCH* | Tracy, S | 2004 | 309 | 14.71(52) |
| 21 | RNAi-Mediated Silencing of Nuclear Factor Erythroid-2–Related Factor 2 Gene Expression in Non–Small Cell Lung Cancer Inhibits Tumor Growth and Increases Efficacy of Chemotherapy | *CANCER RESEARCH* | Singh, A | 2008 | 300 | 17.65(36) |
| 22 | An NQO1- and PARP-1-mediated cell death pathway induced in non-small-cell lung cancer cells by β-lapachone | *PROCEEDINGS OF THE NATIONAL ACADEMY OF SCIENCES OF THE UNITED STATES OF AMERICA* | Bey, EA | 2007 | 287 | 15.94(41) |
| 23 | Heterodimerization of Insulin-like Growth Factor Receptor/Epidermal Growth Factor Receptor and Induction of Survivin Expression Counteract the Antitumor Action of Erlotinib | *CANCER RESEARCH* | Morgillo, F | 2006 | 287 | 15.11(45) |
| 24 | Gefitinib-Induced Killing of NSCLC Cell Lines Expressing Mutant EGFR Requires BIM and Can Be Enhanced by BH3 Mimetics | *PLOS MEDICINE* | Cragg, MS | 2007 | 279 | 15.50(43) |
| 25 | Dasatinib (BMS-354825) Tyrosine Kinase Inhibitor Suppresses Invasion and Induces Cell Cycle Arrest and Apoptosis of Head and Neck Squamous Cell Carcinoma and Non–Small Cell Lung Cancer Cells | *CLINICAL CANCER RESEARCH* | Johnson, FM | 2005 | 269 | 13.45(55) |
| 26 | MicroRNA-451 functions as a tumor suppressor in human non-small cell lung cancer by targeting ras-related protein 14 (RAB14) | *ONCOGENE* | Wang, R | 2011 | 268 | 19.14(30) |
| 27 | Nelfinavir, A Lead HIV Protease Inhibitor, Is a Broad-Spectrum, Anticancer Agent that Induces Endoplasmic Reticulum Stress, Autophagy, and Apoptosis In vitro and In vivo | *CLINICAL CANCER RESEARCH* | Gills, J | 2007 | 268 | 14.89(48) |
| 28 | Lung Cancer Cell Lines Harboring MET Gene Amplification Are Dependent on Met for Growth and Survival | *CANCER RESEARCH* | Lutterbach, B | 2007 | 267 | 14.83(50) |
| 29 | Differential Effects of Gefitinib and Cetuximab on Non–small-cell Lung Cancers Bearing Epidermal Growth Factor Receptor Mutations | *JNCI-JOURNAL OF THE NATIONAL CANCER INSTITUTE* | Mukohara, T | 2005 | 267 | 13.35(58) |
| 30 | HGS-ETR1, a fully human TRAIL-receptor 1 monoclonal antibody, induces cell death in multiple tumour types in vitro and in vivo | *BRITISH JOURNAL OF CANCER* | Pukac, L | 2005 | 257 | 12.85(61) |
| 31 | Differential induction of apoptosis in HER2 and EGFR addicted cancers following PI3K inhibition | *PROCEEDINGS OF THE NATIONAL ACADEMY OF SCIENCES OF THE UNITED STATES OF AMERICA* | Faber, AC | 2009 | 254 | 15.88(42) |
| 32 | Nicotine inhibits apoptosis induced by chemotherapeutic drugs by up-regulating XIAP and survivin | *PROCEEDINGS OF THE NATIONAL ACADEMY OF SCIENCES OF THE UNITED STATES OF AMERICA* | Dasgupta, P | 2006 | 254 | 13.37(57) |
| 33 | hsa_circ_0013958: a circular RNA and potential novel biomarker for lung adenocarcinoma | *FEBS JOURNAL* | Zhu, XL | 2017 | 253 | 31.63(14) |
| 34 | Combined Targeting of the Estrogen Receptor and the Epidermal Growth Factor Receptor in Non–Small Cell Lung Cancer Shows Enhanced Antiproliferative Effects | *CANCER RESEARCH* | Stabile, LP | 2005 | 249 | 12.45(63) |
| 35 | Tobacco components stimulate Akt-dependent proliferation and NFκB-dependent survival in lung cancer cells | *CARCINOGENESIS* | Tsurutani, J | 2005 | 248 | 12.40(64) |
| 36 | A critical role for the long non-coding RNA GAS5 in proliferation and apoptosis in non-small-cell lung cancer | *MOLECULAR CARCINOGENESIS* | Shi, XF | 2015 | 244 | 24.40(21) |
| 37 | miR-181a and miR-630 Regulate Cisplatin-Induced Cancer Cell Death | *CANCER RESEARCH* | Galluzzi, L | 2010 | 240 | 16.00(39) |
| 38 | Death Receptor Regulation and Celecoxib-Induced Apoptosis in Human Lung Cancer Cells | *JNCI-JOURNAL OF THE NATIONAL CANCER INSTITUTE* | Liu, XG | 2004 | 240 | 11.43(70) |
| 39 | An Alternative Inhibitor Overcomes Resistance Caused by a Mutation of the Epidermal Growth Factor Receptor | *CANCER RESEARCH* | Kobayashi, S | 2005 | 228 | 11.40(72) |
| 40 | MicroRNA signatures of TRAIL resistance in human non-small cell lung cancer | *ONCOGENE* | Garofalo, M | 2008 | 227 | 13.35(59) |
| 41 | miR-146a Inhibits Cell Growth, Cell Migration and Induces Apoptosis in Non-Small Cell Lung Cancer Cells | *PLOS ONE* | Chen, G | 2013 | 224 | 18.67(31) |
| 42 | Curcumin Induces Apoptosis in Human Non-small Cell Lung Cancer NCI-H460 Cells through ER Stress and Caspase Cascade- and Mitochondria-dependent Pathways | *ANTICANCER RESEARCH* | Wu, SH | 2010 | 223 | 14.87(49) |
| 43 | Metformin inhibits growth and enhances radiation response of non-small cell lung cancer (NSCLC) through ATM and AMPK | *BRITISH JOURNAL OF CANCER* | Storozhuk, Y | 2013 | 222 | 18.50(33) |
| 44 | Inhibition of Wnt-2-mediated signaling induces programmed cell death in non-small-cell lung cancer cells | *ONCOGENE* | You, L | 2004 | 218 | 10.38(84) |
| 45 | Novel peptidomimetic inhibitors of signal transducer and activator of transcription 3 dimerization and biological activity | *MOLECULAR CANCER THERAPEUTICS* | Turkson, J | 2004 | 218 | 10.38(85) |
| 46 | Epidermal Growth Factor Receptors Harboring Kinase Domain Mutations Associate with the Heat Shock Protein 90 Chaperone and Are Destabilized following Exposure to Geldanamycins | *CANCER RESEARCH* | Shimamura, T | 2005 | 217 | 10.85(77) |
| 47 | Identifying genotype-dependent efficacy of single and combined PI3K- and MAPK-pathway inhibition in cancer | *PROCEEDINGS OF THE NATIONAL ACADEMY OF SCIENCES OF THE UNITED STATES OF AMERICA* | Sos, ML | 2009 | 215 | 13.44(56) |
| 48 | A Monoclonal Antibody against Wnt-1 Induces Apoptosis in Human Cancer Cells | *NEOPLASIA* | He, B | 2004 | 214 | 10.19(87) |
| 49 | Implication of the Insulin-like Growth Factor-IR Pathway in the Resistance of Non–small Cell Lung Cancer Cells to Treatment with Gefitinib | *CLINICAL CANCER RESEARCH* | Morgillo, F | 2007 | 212 | 11.78(67) |
| 50 | Antitumor effect of β-elemene in non-small-cell lung cancer cells is mediated via induction of cell cycle arrest and apoptotic cell death | *CELLULAR AND MOLECULAR LIFE SCIENCES* | Wang, G | 2005 | 211 | 10.55(82) |
| 51 | Long Noncoding RNA PVT1 Promotes Non–Small Cell Lung Cancer Cell Proliferation through Epigenetically Regulating LATS2 Expression | *MOLECULAR CANCER THERAPEUTICS* | Wan, L | 2016 | 208 | 23.11(24) |
| 52 | Treatment-Induced Tumor Dormancy through YAP-Mediated Transcriptional Reprogramming of the Apoptotic Pathway | *CANCER CELL* | Kurppa, KJ | 2020 | 205 | 41.00(5) |
| 53 | EZH2-mediated epigenetic suppression of long noncoding RNA SPRY4-IT1 promote s NSCLC cell proliferation and metastasis by affecting the epithelial–mesenchymal transition | *CELL DEATH & DISEASE* | Sun, M | 2014 | 203 | 18.45(34) |
| 54 | Signal Transducer and Activator of Transcription 3 Is Required for the Oncogenic Effects of Non–Small-Cell Lung Cancer–Associated Mutations of the Epidermal Growth Factor Receptor | *CANCER RESEARCH* | Alvarez, JV | 2006 | 201 | 10.58(81) |
| 55 | Activated Epidermal Growth Factor Receptor–Stat-3 Signaling Promotes Tumor Survival In vivo in Non–Small Cell Lung Cancer | *CLINICAL CANCER RESEARCH* | Haura, EB | 2005 | 199 | 9.95(90) |
| 56 | Circular RNA 100146 functions as an oncogene through direct binding to miR-361-3p and miR-615-5p in non-small cell lung cancer | *MOLECULAR CANCER* | Chen, LJ | 2019 | 197 | 32.83(12) |
| 57 | Small nucleolar RNA 42 acts as an oncogene in lung tumorigenesis | *ONCOGENE* | Mei, YP | 2012 | 197 | 15.15(44) |
| 58 | Class III β-Tubulin Mediates Sensitivity to Chemotherapeutic Drugs in Non–Small Cell Lung Cancer | *CANCER RESEARCH* | Gan, PP | 2007 | 195 | 10.83(78) |
| 59 | Generation and Characterisation of Cisplatin-Resistant Non-Small Cell Lung Cancer Cell Lines Displaying a Stem-Like Signature | *PLOS ONE* | Barr, MP | 2013 | 192 | 16.00(40) |
| 60 | Nicotine Inactivation of the Proapoptotic Function of Bax through Phosphorylation | *JOURNAL OF BIOLOGICAL CHEMISTRY* | Xin, MG | 2005 | 191 | 9.55(91) |
| 61 | Proliferative inhibition, cell-cycle dysregulation, and induction of apoptosis by ursolic acid in human non-small cell lung cancer A549 cells | *LIFE SCIENCES* | Hsu, YL | 2004 | 191 | 9.10(95) |
| 62 | Downregulation of GSDMD attenuates tumor proliferation via the intrinsic mitochondrial apoptotic pathway and inhibition of EGFR/Akt signaling and predicts a good prognosis in non‑small cell lung cancer | *ONCOLOGY REPORTS* | Gao, JW | 2018 | 189 | 27.00(17) |
| 63 | Mcl-1 regulates survival and sensitivity to diverse apoptotic stimuli in human non-small cell lung cancer cells | *CANCER BIOLOGY & THERAPY* | Song, LX | 2005 | 183 | 9.15(94) |
| 64 | Schedule-Dependent Cytotoxic Synergism of Pemetrexed and Erlotinib in Human Non–Small Cell Lung Cancer Cells | *CLINICAL CANCER RESEARCH* | Li, TH | 2007 | 182 | 10.11(88) |
| 65 | Polyphyllin VI Induces Caspase-1-Mediated Pyroptosis via the Induction of ROS/NF-κB/NLRP3/GSDMD Signal Axis in Non-Small Cell Lung Cancer | *CANCERS* | Teng, JF | 2020 | 180 | 36.00(7) |
| 66 | Downregulation of BRAF activated non-coding RNA is associated with poor prognosis for non-small cell lung cancer and promotes metastasis by affecting epithelial-mesenchymal transition | *MOLECULAR CANCER* | Sun, M | 2014 | 178 | 16.18(37) |
| 67 | Nanoscaled Poly(l-glutamic acid)/Doxorubicin-Amphiphile Complex as pH-responsive Drug Delivery System for Effective Treatment of Nonsmall Cell Lung Cancer | *ACS APPLIED MATERIALS & INTERFACES* | Li, MQ | 2013 | 178 | 14.83(51) |
| 68 | Circular RNA circ-CPA4/ let-7 miRNA/PD-L1 axis regulates cell growth, stemness, drug resistance and immune evasion in non-small cell lung cancer (NSCLC) | *JOURNAL OF EXPERIMENTAL & CLINICAL CANCER RESEARCH* | Hong, WJ | 2020 | 177 | 35.40(8) |
| 69 | Exosomal formulation enhances therapeutic response of celastrol against lung cancer | *EXPERIMENTAL AND MOLECULAR PATHOLOGY* | Aqil, F | 2016 | 177 | 19.67(29) |
| 70 | Cathepsin B Mediates Caspase-Independent Cell Death Induced by Microtubule Stabilizing Agents in Non-Small Cell Lung Cancer Cells | *CANCER RESEARCH* | Bröker, LE | 2004 | 177 | 8.34(97) |
| 71 | Gallic Acid Induces Apoptosis via Caspase-3 and Mitochondrion-Dependent Pathways in Vitro and Suppresses Lung Xenograft Tumor Growth in Vivo | *JOURNAL OF AGRICULTURAL AND FOOD CHEMISTRY* | Ji, BC | 2009 | 176 | 11.00(75) |
| 72 | Combined Radiotherapy and Anti–PD-L1 Antibody Synergistically Enhances Antitumor Effect in Non–Small Cell Lung Cancer | *JOURNAL OF THORACIC ONCOLOGY* | Gong, XM | 2017 | 175 | 21.88(26) |
| 73 | Monitoring Tumor Glucose Utilization by Positron Emission Tomography for the Prediction of Treatment Response to Epidermal Growth Factor Receptor Kinase Inhibitors | *CLINICAL CANCER RESEARCH* | Su, H | 2006 | 175 | 9.21(92) |
| 74 | Chemogenomic Profiling Provides Insights into the Limited Activity of Irreversible EGFR Inhibitors in Tumor Cells Expressing the T790M EGFR Resistance Mutation | *CANCER RESEARCH* | Sos, ML | 2010 | 173 | 11.53(68) |
| 75 | Thymoquinone and cisplatin as a therapeutic combination in lung cancer: In vitro and in vivo | *JOURNAL OF EXPERIMENTAL & CLINICAL CANCER RESEARCH* | Jafri, SH | 2010 | 172 | 11.47(69) |
| 76 | Hierarchical involvement of Bak, VDAC1 and Bax in cisplatin-induced cell death | *ONCOGENE* | Tajeddine, N | 2008 | 170 | 10.00(89) |
| 77 | MicroRNA-7 Inhibits the Growth of Human Non-Small Cell Lung Cancer A549 Cells through Targeting BCL-2 | *INTERNATIONAL JOURNAL OF BIOLOGICAL SCIENCES* | Xiong, SD | 2011 | 167 | 11.93(66) |
| 78 | An anti-Axl monoclonal antibody attenuates xenograft tumor growth and enhances the effect of multiple anticancer therapies | *ONCOGENE* | Ye, X | 2010 | 167 | 11.13(74) |
| 79 | Long Intergenic Noncoding RNA 00511 Acts as an Oncogene in Non–small-cell Lung Cancer by Binding to EZH2 and Suppressing p57 | *MOLECULAR THERAPY-NUCLEIC ACIDS* | Sun, CC | 2016 | 166 | 18.44(35) |
| 80 | Antitumor Effects of a Novel Chromosome Region Maintenance 1 (CRM1) Inhibitor on Non-Small Cell Lung Cancer Cells In Vitro and in Mouse Tumor Xenografts | *PLOS ONE* | Wang, S | 2014 | 166 | 15.09(46) |
| 81 | Enhanced antitumor efficacy by Paclitaxel-loaded Pluronic P123/F127 mixed micelles against non-small cell lung cancer based on passive tumor targeting and modulation of drug resistance | *EUROPEAN JOURNAL OF PHARMACEUTICS AND BIOPHARMACEUTICS* | Wei, Z | 2010 | 165 | 11.00(76) |
| 82 | Human lung cancer cells express functionally active Toll-like receptor 9 | *RESPIRATORY RESEARCH* | Droemann, D | 2005 | 165 | 8.25(98) |
| 83 | Cyclooxygenase (COX)-2 Inhibitor Celecoxib Abrogates TNF-Induced NF-κB Activation through Inhibition of Activation of IκBα Kinase and Akt in Human Non-Small Cell Lung Carcinoma: Correlation with Suppression of COX-2 Synthesis | *JOURNAL OF IMMUNOLOGY* | Shishodia, S | 2004 | 164 | 7.81(99) |
| 84 | Extracellular vesicles secreted by hypoxia pre-challenged mesenchymal stem cells promote non-small cell lung cancer cell growth and mobility as well as macrophage M2 polarization via miR-21-5p delivery | *JOURNAL OF EXPERIMENTAL & CLINICAL CANCER RESEARCH* | Ren, WH | 2019 | 163 | 27.17(16) |
| 85 | Curcumin promotes apoptosis by activating the p53-miR-192-5p/215-XIAP pathway in non-small cell lung cancer | *CANCER LETTERS* | Ye, MX | 2015 | 161 | 16.10(38) |
| 86 | Phospho-Akt Overexpression in Non–Small Cell Lung Cancer Confers Significant Stage-Independent Survival Disadvantage | *CLINICAL CANCER RESEARCH* | David, O | 2004 | 161 | 7.67(100) |
| 87 | MicroRNA-21 (miR-21) Regulates Cellular Proliferation, Invasion, Migration, and Apoptosis by Targeting PTEN, RECK and Bcl-2 in Lung Squamous Carcinoma, Gejiu City, China | *PLOS ONE* | Xu, LF | 2014 | 160 | 14.55(53) |
| 88 | Targeting p21-activated kinase 1 (PAK1) to induce apoptosis of tumor cells | *PROCEEDINGS OF THE NATIONAL ACADEMY OF SCIENCES OF THE UNITED STATES OF AMERICA* | Ong, CC | 2011 | 160 | 11.43(71) |
| 89 | Chronic cisplatin treatment promotes enhanced damage repair and tumor progression in a mouse model of lung cancer | *GENES & DEVELOPMENT* | Oliver, TG | 2010 | 159 | 10.60(80) |
| 90 | Epithelial NF-κB activation promotes urethane-induced lung carcinogenesis | *PROCEEDINGS OF THE NATIONAL ACADEMY OF SCIENCES OF THE UNITED STATES OF AMERICA* | Stathopoulos, GT | 2007 | 159 | 8.83(96) |
| 91 | Coxsackievirus B3 Is an Oncolytic Virus with Immunostimulatory Properties That Is Active against Lung Adenocarcinoma | *CANCER RESEARCH* | Miyamoto, S | 2012 | 158 | 12.15(65) |
| 92 | Overexpression of the JmjC histone demethylase KDM5B in human carcinogenesis: involvement in the proliferation of cancer cells through the E2F/RB pathway | *MOLECULAR CANCER* | Hayami, S | 2010 | 157 | 10.47(83) |
| 93 | The Noncoding RNA Expression Profile and the Effect of lncRNA AK126698 on Cisplatin Resistance in Non-Small-Cell Lung Cancer Cell | *PLOS ONE* | Yang, Y | 2013 | 156 | 13.00(60) |
| 94 | Sphingosine Kinase-1 Enhances Resistance to Apoptosis through Activation of PI3K/Akt/NF-κB Pathway in Human Non–Small Cell Lung Cancer | *CLINICAL CANCER RESEARCH* | Song, LB | 2011 | 156 | 11.14(73) |
| 95 | Targeting of AKT1 enhances radiation toxicity of human tumor cells by inhibiting DNA-PKcs-dependent DNA double-strand break repair | *MOLECULAR CANCER THERAPEUTICS* | Toulany, M | 2008 | 156 | 9.18(93) |
| 96 | Gain of Nrf2 Function in Non-Small-Cell Lung Cancer Cells Confers Radioresistance | *ANTIOXIDANTS & REDOX SIGNALING* | Singh, A | 2010 | 155 | 10.33(86) |
| 97 | Rhamnetin and Cirsiliol Induce Radiosensitization and Inhibition of Epithelial-Mesenchymal Transition (EMT) by miR-34a-mediated Suppression of Notch-1 Expression in Non-small Cell Lung Cancer Cell Lines | *JOURNAL OF BIOLOGICAL CHEMISTRY* | Kang, J | 2013 | 151 | 12.58(62) |
| 98 | miR-340 inhibits tumor cell proliferation and induces apoptosis by targeting multiple negative regulators of p27 in non-small cell lung cancer | *ONCOGENE* | Fernandez, S | 2015 | 150 | 15.00(47) |
| 99 | Targeting SOD1 reduces experimental non–small-cell lung cancer | *JOURNAL OF CLINICAL INVESTIGATION* | Glasauer, A | 2014 | 150 | 13.64(54) |
| 100 | Non-redox-active lipoate derivates disrupt cancer cell mitochondrial metabolism and are potent anticancer agents in vivo | *JOURNAL OF MOLECULAR MEDICINE-JMM* | Zachar, Z | 2011 | 150 | 10.71(79) |

**Table S2**. The 45 journals ranked by the number of articles.

| Rank | Journal | Number of articles | Total citation  (Rank) | JIF(2023)  (Rank) | JCI(2023) | JCR(2023) |
| --- | --- | --- | --- | --- | --- | --- |
| 1 | *CANCER RESEARCH* | 15 | 3,691(1) | 12.5(10) | 1.99 | Q1 |
| 2 | *CLINICAL CANCER RESEARCH* | 9 | 2,403(2) | 10.4(13) | 2.52 | Q1 |
| 3 | *ONCOGENE* | 7 | 1,397(4) | 6.9(23) | 1.47 | Q1 |
| 4 | *PLOS ONE* | 6 | 1,249(6) | 2.9(43) | 0.88 | Q1 |
| 5 | *PROCEEDINGS OF THE NATIONAL ACADEMY OF SCIENCES OF THE UNITED STATES OF AMERICA* | 6 | 1,329(7) | 9.4(15) | 2.40 | Q1 |
| 6 | *CANCER CELL* | 4 | 1,603(3) | 48.8(3) | 7.57 | Q1 |
| 7 | *MOLECULAR CANCER THERAPEUTICS* | 4 | 922(9) | 5.4(30) | 1.18 | Q1 |
| 8 | *JOURNAL OF EXPERIMENTAL & CLINICAL CANCER RESEARCH* | 3 | 512(15) | 11.4(11) | 2.35 | Q1 |
| 9 | *MOLECULAR CANCER* | 3 | 532(14) | 27.7(5) | 5.25 | Q1 |
| 10 | *BRITISH JOURNAL OF CANCER* | 2 | 479(17) | 6.4(24) | 1.46 | Q1 |
| 11 | *NATURE MEDICINE* | 2 | 922(10) | 58.7(1) | 13.63 | Q1 |
| 12 | *NATURE* | 2 | 1,006(8) | 50.5(2) | 11.30 | Q1 |
| 13 | *JOURNAL OF BIOLOGICAL CHEMISTRY* | 2 | 342(20) | 4.0(37) | 0.85 | Q2 |
| 14 | *JNCI-JOURNAL OF THE NATIONAL CANCER INSTITUTE* | 2 | 507(16) | 10.0(14) | 2.18 | Q1 |
| 15 | *CELL DEATH & DISEASE* | 2 | 547(13) | 8.1(21) | 1.24 | Q1 |
| 16 | *PLOS MEDICINE* | 2 | 688(12) | 10.5(12) | 2.57 | Q1 |
| 17 | *RESPIRATORY RESEARCH* | 1 | 165(39) | 4.7(33) | 1.27 | Q1 |
| 18 | *ONCOLOGY REPORTS* | 1 | 189(29) | 3.8(38) | 0.85 | Q2 |
| 19 | *NEOPLASIA* | 1 | 214(26) | 6.3(25) | 1.44 | Q1 |
| 20 | *NATURE CELL BIOLOGY* | 1 | 393(18) | 17.3(7) | 2.94 | Q1 |
| 21 | *MOLECULAR THERAPY-NUCLEIC ACIDS* | 1 | 166(37) | 8.8(17) | 2.08 | Q1 |
| 22 | *MOLECULAR CARCINOGENESIS* | 1 | 244(24) | 3.0(42) | 0.80 | Q2 |
| 23 | *LIFE SCIENCES* | 1 | 191(28) | 5.2(31) | 1.39 | Q1 |
| 24 | *JOURNAL OF THORACIC ONCOLOGY* | 1 | 175(35) | 21.1(6) | 4.29 | Q1 |
| 25 | *JOURNAL OF MOLECULAR MEDICINE-JMM* | 1 | 150(45) | 4.8(32) | 0.93 | Q1 |
| 26 | *JOURNAL OF IMMUNOLOGY* | 1 | 164(40) | 3.6(39) | 0.73 | Q2 |
| 27 | *JOURNAL OF CLINICAL INVESTIGATION* | 1 | 150(44) | 13.3(9) | 3.45 | Q1 |
| 28 | *JOURNAL OF AGRICULTURAL AND FOOD CHEMISTRY* | 1 | 176(34) | 5.7(28) | 1.33 | Q1 |
| 29 | *INTERNATIONAL JOURNAL OF BIOLOGICAL SCIENCES* | 1 | 167(36) | 8.2(19) | 1.87 | Q1 |
| 30 | *GENES & DEVELOPMENT* | 1 | 159(42) | 7.5(22) | 1.87 | Q1 |
| 31 | *FEBS JOURNAL* | 1 | 253(22) | 5.5(29) | 0.79 | Q1 |
| 32 | *EXPERIMENTAL AND MOLECULAR PATHOLOGY* | 1 | 177(33) | 2.8(44) | 1.10 | Q2 |
| 33 | *EUROPEAN JOURNAL OF PHARMACEUTICS AND BIOPHARMACEUTICS* | 1 | 165(38) | 4.4(35) | 1.41 | Q1 |
| 34 | *CURRENT BIOLOGY* | 1 | 910(11) | 8.1(20) | 1.59 | Q1 |
| 35 | *CELLULAR AND MOLECULAR LIFE SCIENCES* | 1 | 211(27) | 6.2(26) | 0.94 | Q1 |
| 36 | *CELL DEATH AND DIFFERENTIATION* | 1 | 326(21) | 13.7(8) | 1.97 | Q1 |
| 37 | *CARCINOGENESIS* | 1 | 248(23) | 3.3(41) | 0.91 | Q2 |
| 38 | *CANCERS* | 1 | 180(31) | 4.5(34) | 0.91 | Q1 |
| 39 | *CANCER LETTERS* | 1 | 161(41) | 9.1(16) | 1.99 | Q1 |
| 40 | *CANCER BIOLOGY & THERAPY* | 1 | 183(30) | 4.4(36) | 0.85 | Q2 |
| 41 | *BMC CANCER* | 1 | 358(19) | 3.4(40) | 0.79 | Q2 |
| 42 | *ANTIOXIDANTS & REDOX SIGNALING* | 1 | 155(43) | 5.9(27) | 1.20 | Q1 |
| 43 | *ANTICANCER RESEARCH* | 1 | 223(25) | 1.6(45) | 0.42 | Q4 |
| 44 | *ACS APPLIED MATERIALS & INTERFACES* | 1 | 178(32) | 8.5(18) | 1.41 | Q1 |
| 45 | *SCIENCE* | 1 | 1,389(5) | 44.8(4) | 9.90 | Q1 |

**Table S3**. List of the top 10 most cited apoptosis studies in NSCLC (from 2021 to March 8, 2024).

| Rank | Title | Journal | First author | Year | Total citation  (WoS Core) | Citation/Year  (Rank) |
| --- | --- | --- | --- | --- | --- | --- |
| 1 | MicroRNA-567 inhibits cell proliferation and induces cell apoptosis in A549 NSCLC cells by regulating cyclin-dependent kinase 8 | *SAUDI JOURNAL OF BIOLOGICAL SCIENCES* | Elkady, MA | 2021 | 74 | 18.50(1) |
| 2 | Apatinib triggers autophagic and apoptotic cell death via VEGFR2/STAT3/PD-L1 and ROS/Nrf2/p62 signaling in lung cancer | *JOURNAL OF EXPERIMENTAL & CLINICAL CANCER RESEARCH* | Xie, CF | 2021 | 70 | 17.50(2) |
| 3 | Auranofin reveals therapeutic anticancer potential by triggering distinct molecular cell death mechanisms and innate immunity in mutant p53 non-small cell lung cancer | *REDOX BIOLOGY* | Boullosa, LF | 2021 | 59 | 14.75(4) |
| 4 | Quercetin induces pro-apoptotic autophagy via SIRT1/AMPK signaling pathway in human lung cancer cell lines A549 and H1299 in vitro | *THORACIC CANCER* | Guo, HJ | 2021 | 57 | 14.25(5) |
| 5 | Tumor-derived exosomal circRNA_102481 contributes to EGFR-TKIs resistance via the miR-30a-5p/ROR1 axis in non-small cell lung cancer | *AGING-US* | Yang, B | 2021 | 52 | 13.00(6) |
| 6 | Long non-coding RNA NEAT1 regulates ferroptosis sensitivity in non-small-cell lung cancer | *JOURNAL OF INTERNATIONAL MEDICAL RESEARCH* | Wu, HX | 2021 | 52 | 13.00(7) |
| 7 | Kaempferol inhibits Nrf2 signalling pathway via downregulation of Nrf2 mRNA and induces apoptosis in NSCLC cells | *ARCHIVES OF BIOCHEMISTRY AND BIOPHYSICS* | Fouzder, C | 2021 | 52 | 13.00(8) |
| 8 | Artemisinin Derivatives Inhibit Non-small Cell Lung Cancer Cells Through Induction of ROS-dependent Apoptosis/Ferroptosis | *JOURNAL OF CANCER* | Zhang, QT | 2021 | 52 | 13.00(9) |
| 9 | Exosome-transmitted circVMP1 facilitates the progression and cisplatin resistance of non-small cell lung cancer by targeting miR-524-5p-METTL3/SOX2 axis | *DRUG DELIVERY* | Xie, HY | 2022 | 50 | 16.67(3) |
| 10 | Small-molecule inhibition of APE1 induces apoptosis, pyroptosis, and necroptosis in non-small cell lung cancer | *CELL DEATH & DISEASE* | Long, KL | 2021 | 44 | 11.00(10) |

**Table S4** Keyword clustering

| Cluster ID | Cluster label | Size | Silhouette | mean(Year) | Label (LLR) |
| --- | --- | --- | --- | --- | --- |
| #0 | lung cancer | 31 | 0.827 | 2009 | lung cancer (9.92, 0.005); phosphorylation (4.87, 0.05); elemene (4.2, 0.05); bax (4.2, 0.05); growth factor (4.2, 0.05) |
| #1 | cancer | 30 | 0.927 | 2012 | cancer (11.51, 0.001); cells (8.07, 0.005); protein (4.53, 0.05); resistance (4.53, 0.05); complex (4.02, 0.05) |
| #2 | e cadherin | 27 | 0.91 | 2011 | e cadherin (10.19, 0.005); repression (10.19, 0.005); differentiation (10.19, 0.005); gencode (5.07, 0.05); family inhibitor (5.07, 0.05) |
| #3 | antioxidant | 18 | 0.851 | 2013 | antioxidant (9.3, 0.005); glutathione (9.3, 0.005); lung neoplasms (4.63, 0.05); caspase-3 (4.63, 0.05); superoxide dismutase 1 sod1 (4.63, 0.05) |
| #4 | breast cancer | 18 | 0.945 | 2009 | breast cancer (8.09, 0.005); chemotherapy (4.64, 0.05); nf kappa b (4.64, 0.05); patient survival (4.08, 0.05); cyclin d1 (4.08, 0.05) |
| #5 | clinical response | 18 | 0.77 | 2006 | clinical response (13.42, 0.001); somatic mutations (8.91, 0.005); tyrosine kinase (5.77, 0.05); egfr mutations (5.32, 0.05); gene mutations (5.32, 0.05) |
| #6 | acquired resistance | 18 | 0.81 | 2006 | acquired resistance (14.25, 0.001); cell lung cancer (7.9, 0.005); gefitinib (6.5, 0.05); egf receptor (5.84, 0.05); akt (5.84, 0.05) |
| #7 | growth | 17 | 0.945 | 2014 | growth (6.29, 0.05); milk-derived exosomes (5.77, 0.05); population (5.77, 0.05); paclitaxel (5.77, 0.05); tead (5.77, 0.05) |
| #8 | induced apoptosis | 16 | 0.816 | 2006 | induced apoptosis (11.16, 0.001); survival (6.63, 0.05); transcription factor (5.12, 0.05); anticancer agents (5.12, 0.05); human lung cancer (5.12, 0.05) |
| #9 | arrest | 10 | 0.868 | 2012 | arrest (11.47, 0.001); identification (6.16, 0.05); retinoblastoma protein (5.7, 0.05); tumor suppressor genes (5.7, 0.05); ovarian cancer (5.7, 0.05) |


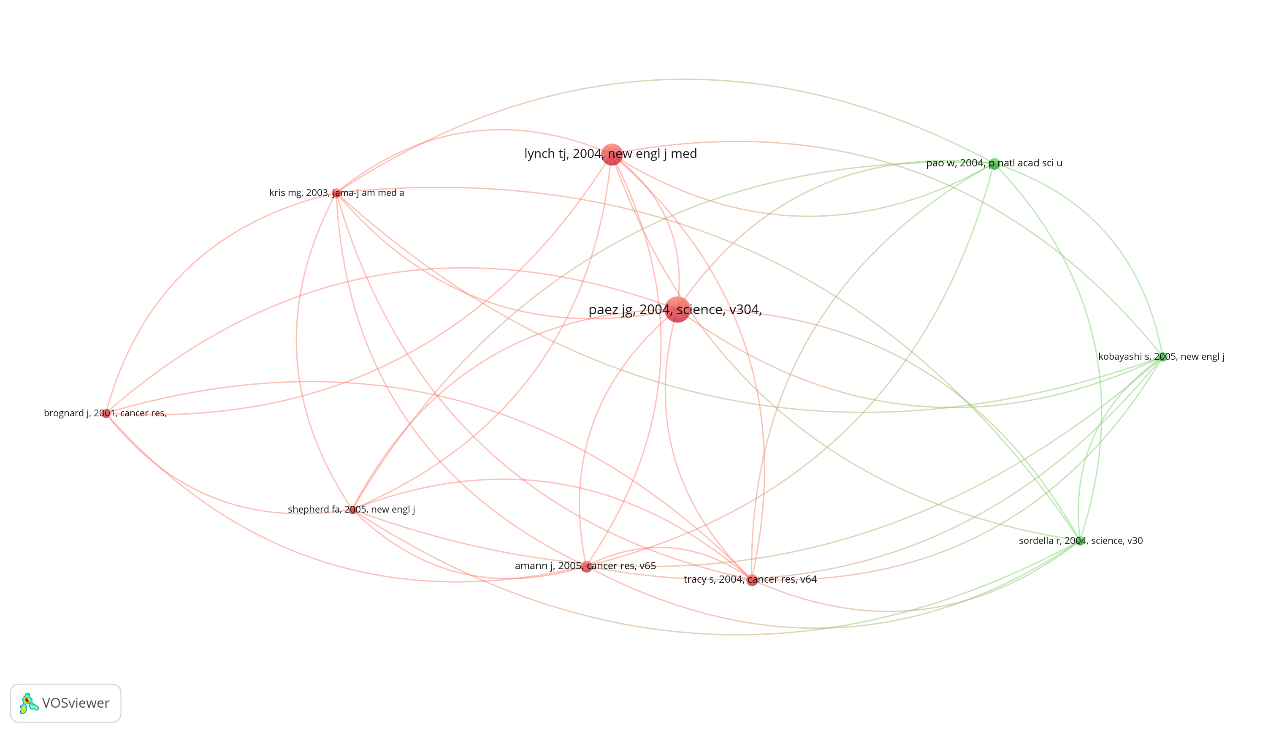
Note: Nodes symbolize individual references, with the size of each node corresponding to the number of citations that particular reference has received. A larger node size denotes a higher frequency of co-citations. Additionally, a line connecting nodes signifies that the references are cited concurrently.

**Figure S1** Co-citation map of references
